# Thioredoxin system dysregulation and calpain activation drive myocardial disulfidptosis via pathological disulfide bonds remodeling

**DOI:** 10.1038/s41420-026-03200-0

**Published:** 2026-06-19

**Authors:** Aling Tang, Bingqing Chen, Weizhen Zhang, Zhimin Gong, Mingming Jin, Xiaoling Xu, Yi Shi, Wei Chen

**Affiliations:** 1 Longhua Hospital Affiliated to Shanghai University of Traditional Chinese Medicine, Shanghai, China; 2 Key Laboratory of Artificial Organs and Computational Medicine of Zhejiang Province, Shulan International Medical College, Zhejiang Shuren University, Hangzhou, China; 3 Yueyang Hospital of Integrated Traditional Chinese and Western Medicine, Shanghai University of Traditional Chinese Medicine, Shanghai, China; 4 Shanghai University of Medicine & Health Sciences, Shanghai, China

**Keywords:** Cell death, Cardiovascular diseases

## Abstract

Myocardial infarction (MI) remains a leading cause of death and disability worldwide, yet the molecular mechanisms underlying cardiomyocyte death during ischemic injury are not fully understood. Here, we identify disulfidptosis—a recently described form of regulated cell death—as a novel contributor to myocardial ischemic injury. In ischemia-mimetic models, glucose and oxygen deprivation lead to NADPH depletion and excessive disulfide bond accumulation in cardiomyocytes, accompanied by F-actin cytoskeletal collapse, a defining feature of disulfidptosis. Mechanistically, impairment of the NADPH/thioredoxin (Trx) system amplifies disulfide stress, while calcium overload–induced activation of calpains disrupts cytoskeletal protein conformation, removing steric constraints that normally prevent aberrant disulfide bonding. The synergistic effect of these two processes creates favorable oxidative and spatial structural conditions for the occurrence of disulfidptosis. Inhibition of Trx activity promotes disulfide death; pharmacological inhibition of calpainthe or blockade of calcium overload significantly reduces disulfide accumulation and preserves cytoskeletal integrity, confirming their crucial role in ischemia-induced disulfidptosis. Based on the above findings, this study confirms that disulfidptosis represents a previously unrecognized mechanism of cardiomyocyte death in myocardial ischemia, revealing the mechanistic link between metabolic stress, redox imbalance, and cytoskeletal collapse; and proposing a novel pathological remodeling process of disulfide bonds characterized by “Cleavage–Fragmentation–Mismatch”. These insights provide new conceptual and therapeutic perspectives for cardioprotection.

**Figure:**
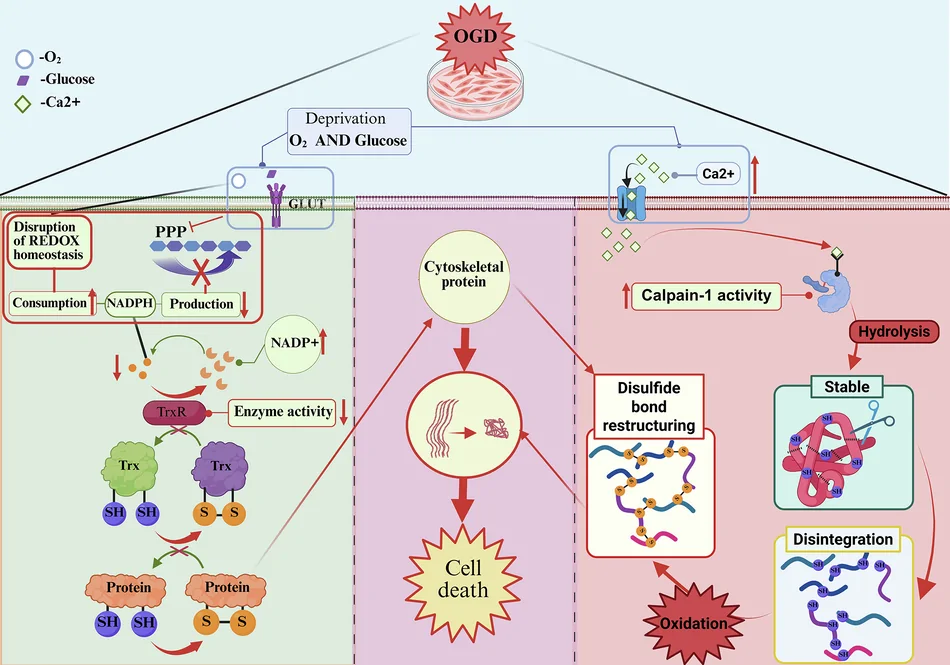

## Introduction

Myocardial infarction (MI) remains one of the leading causes of cardiovascular-related disability and mortality worldwide. In the United States, coronary heart disease (CHD) is the leading cause of cardiovascular mortality, accounting for 39.5% of all cardiovascular disease-related deaths. In 2022 alone, CHD was responsible for 371,506 fatalities [1]. Globally, ischemic heart disease (IHD) is projected to become the most prevalent form of cardiovascular disease (CVD) by 2050. Between 2025 and 2050, mortality attributed to IHD is expected to increase by 93.4% [2]. These findings highlight a growing public health concern and underscore the urgent need to elucidate novel molecular mechanisms underlying myocardial injury.

Myocardial ischemia arises from an imbalance between cardiac metabolic demand and coronary blood supply [3]. When coronary occlusion persists beyond 20–40 min, irreversible cardiomyocyte death ensues [4]. Although classical necrosis and apoptosis have long been recognized as dominant cell death modes in ischemic injury, emerging evidence points to additional regulated pathways that may contribute to cardiomyocyte loss [5, 6]. Recently, a distinct form of regulated cell death—termed disulfidptosis—has been identified. This process is triggered by metabolic stress, such as glucose deprivation, which suppresses the pentose phosphate pathway (PPP) and depletes NADPH. The resulting NADPH deficiency leads to abnormal intracellular disulfide bond accumulation and disulfide stress. Activation of the Rac–WAVE regulatory complex (WRC) axis subsequently induces aberrant disulfide bond formation among actin cytoskeletal proteins, causing F-actin network collapse and cell death [7]. From a metabolic perspective, the healthy heart relies primarily on mitochondrial oxidative phosphorylation for ATP generation. However, during coronary artery occlusion, the interruption of glucose and oxygen supply forces the myocardium to depend on anaerobic glycolysis [8]. This hypoxic, glucose-deprived microenvironment closely resembles the metabolic conditions that precipitate disulfidptosis. Yet, whether disulfidptosis contributes to myocardial ischemic injury remains unexplored.

The initiation of disulfidptosis involves two interconnected molecular events: disulfide bond accumulation resulting from NADPH depletion and subsequent cytoskeletal collapse. Cellular disulfide homeostasis is maintained mainly by two NADPH-dependent reductase systems—the thioredoxin (Trx) and glutaredoxin (Grx) systems [9]. Studies in diabetes and its complications have shown that inhibition of the Trx system exacerbates disulfide stress and accelerates disease progression [10]. However, the specific regulatory roles of these redox systems in cardiomyocyte disulfidptosis remain unknown.

The mechanisms underlying cytoskeletal collapse during disulfidptosis also remain incompletely understood. One key question is why cytoskeletal proteins are especially susceptible to disulfide bond formation. Under physiological conditions, proteins adopt well-ordered folded conformations that remain relatively stable. This structural stability imposes steric hindrance between spatially distant cysteine residues, preventing indiscriminate disulfide bond formation [11]. However, when protein conformation is disrupted, the originally ordered structure becomes disorganized, reducing steric constraints between cysteine residues and thereby providing the structural prerequisites for disulfide bond formation. Numerous proteases in cells specifically cleave peptide bonds, leading to structural loosening of proteins [12]. Among these, calpain—a calcium-dependent protease—targets cytoskeletal proteins as substrates [13]. In myocardial ischemia, calcium overload and calpain activation are hallmark pathological events. Whether calpain may facilitate disulfide bond formation by removing structural barriers remains to be further confirmed [14].

Herein, the present study investigates whether disulfidptosis contributes to myocardial ischemic injury and elucidates its molecular mechanisms. Specifically, we examine the role of NADPH/Trx system dysfunction in promoting disulfide accumulation and explore how calpain-mediated conformational disruption of cytoskeletal proteins facilitates aberrant disulfide bonding. Our findings provide the first evidence that disulfidptosis occurs in cardiomyocytes under ischemic injury-like conditions and uncover a mechanistic link between metabolic stress, disulfide accumulation, and cytoskeletal collapse. These insights not only deepen our understanding of cardiomyocyte death in ischemia but also suggest novel therapeutic strategies for myocardial infarction.

This study first confirms that disulfidptosis is involved in myocardial infarction-induced cardiomyocyte injury and further investigates its molecular mechanisms, including NADPH depletion, dysregulation of the Trx system, and increased fragility of the cytoskeletal network due to abnormal activation of calpains.

## Results

### Disulfidptosis mediates OGD-induced cardiomyocyte death

**Fig. 1:**
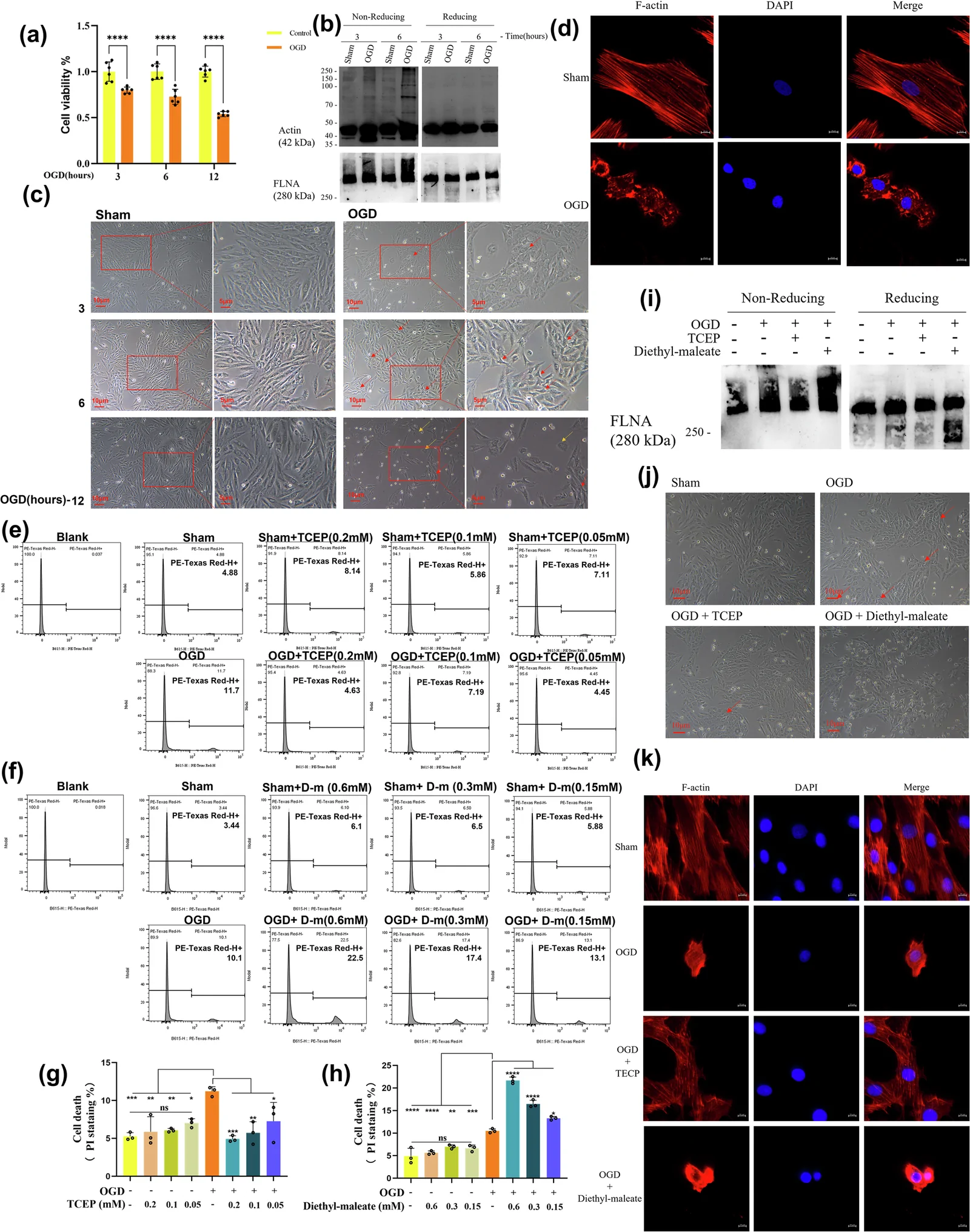
Disulfidptosis mediates OGD-induced cardiomyocyte death. **a** CCK-8 assay showing cell viability at 3, 6, and 12 h of OGD. **b** Western blot analysis of reduced and non-reduced Actin and FLNA in sham and OGD-treated H9C2 cells (3 and 6 h). **c** Bright-field images of cells at 200× magnification following 3, 6, and 12 h of OGD. Red arrows indicate cellular morphological contractions; yellow arrows denote dead cells. **d** Laser scanning confocal images showing F-actin fluorescence staining in cells following 6 h of OGD. Detection of cell death in H9C2 cells treated with Sham, Sham+TCEP (0.2 mM, 0.1 mM, 0.05 mM), OGD, and OGD + TCEP (0.2 mM, 0.1 mM, 0.05 mM) for 6 h using PI staining (**e**) Flow cytometric histograms (**g**) Proportion of PI-positive cells. Detection of cell death in H9C2 cells treated with Sham, Sham + Diethylmaleate (0.6 mM, 0.3 mM, 0.15 mM), OGD, and OGD+Diethylmaleate (0.6 mM, 0.3 mM, 0.15 mM) for 6 h using PI staining (**f**) Flow cytometric histograms (**h**) Proportion of PI-positive cells. **i** Western blot analysis of reduced and non-reduced FLNA in H9C2 cells treated with Sham, OGD, OGD + TCEP, and OGD+Diethylmaleate for 6 h. **j** Bright-field images of H9C2 cells treated with Sham, OGD, OGD + TCEP (0.1 mM), and OGD+Diethylmaleate (0.3 mM) for 6 h at 200× magnification; cellular morphological contractions are marked by red arrows. **k** Laser scanning confocal images of H9C2 cells treated with Sham, OGD, OGD + TCEP (0.1 mM), and OGD+Diethylmaleate (0.3 mM) for 6 h. Data represent 3 independent replicates (6 replicates for **a**). Two-way ANOVA with Tukey’s post hoc test was performed for (**a**). One-way ANOVA with Tukey’s post hoc test was performed for (**g**, **h**).

We initially examined whether oxygen-glucose deprivation (OGD) induces disulfidptosis in cardiomyocytes. CCK-8 cell viability assays showed a sustained decrease in cardiomyocyte viability following OGD (Fig. 1a). Cell morphology changes became more pronounced after 6 h of OGD. By 12 h of OGD, cell morphology was significantly disrupted, with nuclear fragmentation indicating cell death. Cytoskeletal proteins (Actin and FLNA) exhibited migration delay under non-reducing conditions, which was absent under reducing conditions, indicating extensive disulfide bond formation between these proteins (Fig. 1b). Bright-field images revealed reduced cell density and noticeable cell shrinkage after 3 hg of OGD, without significant nuclear damage (Fig. 1c). F-actin fluorescence staining indicated significant cytoskeletal contraction, while the nucleus remained intact (Fig. 1d). OGD-induced cell death in H9C2 cells is linked to actin network collapse, driven by disulfide bond formation between cytoskeletal proteins. We further investigated the role of disulfide bonds. The reducing agent TCEP effectively cleaves disulfide bonds, thereby converting extracellular cystine to cysteine in the culture medium, while diethyl maleate, a thiol oxidizer, promotes disulfide bond formation. TCEP significantly reduced OGD-induced cell death (Fig. 1e, g), whereas diethyl maleate markedly increased OGD-induced cell death (Fig. 1f, h). Western blot analysis indicated that TCEP reduced FLNA disulfide bond formation, while diethyl maleate enhanced FLNA disulfide bonding (Fig. 1i). TCEP mitigated cellular deformation and cytoskeletal collapse, whereas diethyl maleate induced significant morphological changes and cytoskeletal contraction (Fig. 1j, k).

### Cystine and cardiomyocyte disulfidptosis

**Fig. 2:**
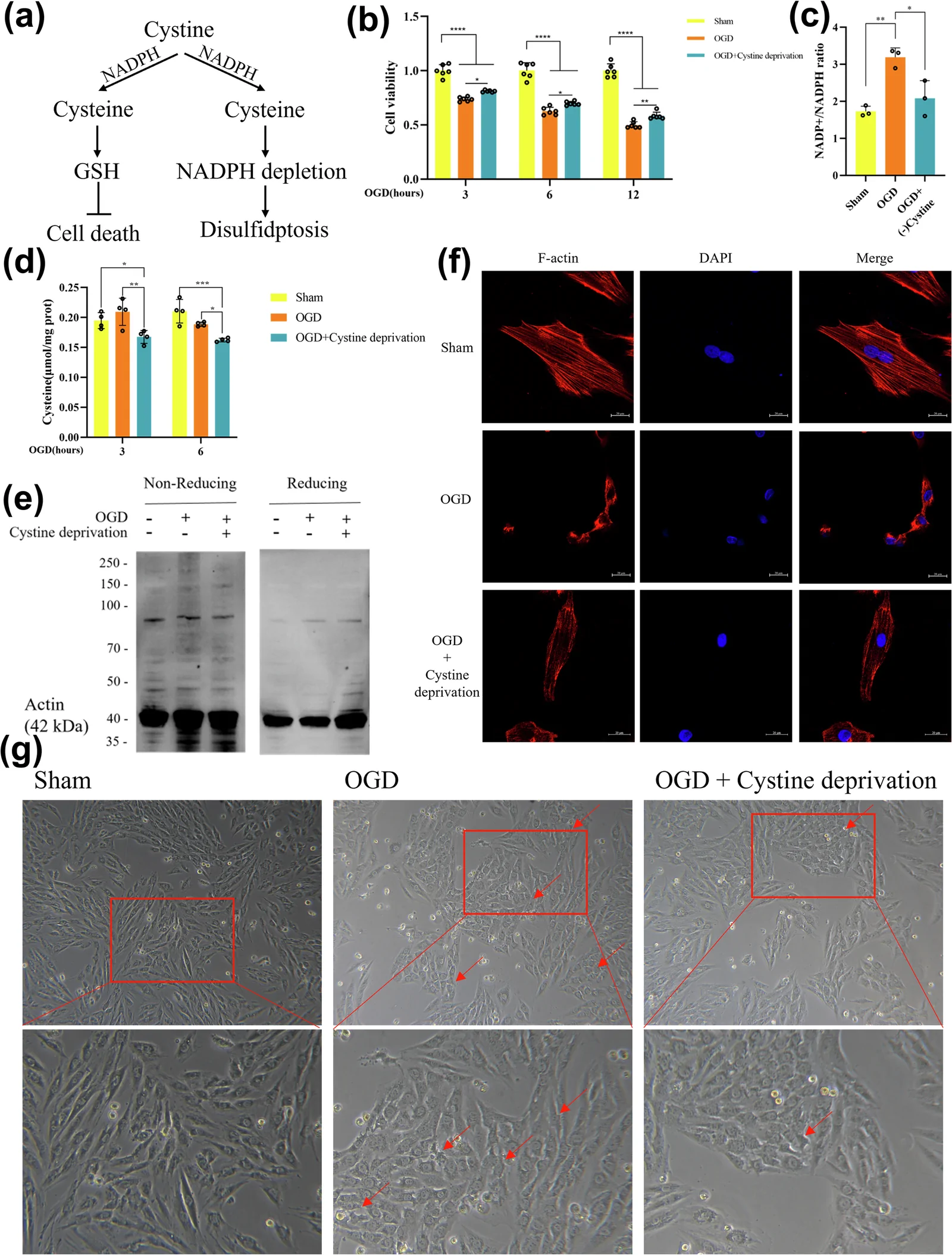
Cystine and cardiomyocyte disulfidptosis. **a** Diagram illustrating the roles of cystine, cysteine, and NADPH in GSH hantioxidant activity and disulfidptosis. **b** CCK-8 assay measuring cell viability in H9C2 cells treated with Sham, OGD, and OGD + cystine deprivation for 3, 6, and 12 h. **c** NADP+/NADPH ratio in H9C2 cells treated with Sham, OGD, and OGD + cystine deprivation for 6 h. **d** Cystine concentration in H9C2 cells treated with Sham, OGD, and OGD + cystine deprivation for 3 and 6 h. **e** Western blot analysis of reduced and non-reduced Actin in H9C2 cells treated with Sham, OGD, and OGD + cystine deprivation for 6 h. **f** Laser scanning confocal images showing F-actin fluorescence staining in H9C2 cells treated with Sham, OGD, and OGD+cystine deprivation for 6 h. **g** Bright-field images of H9C2 cells treated with Sham, OGD, and OGD+cystine deprivation for 6 h, captured at 200× magnification; red arrows indicate cellular morphology contraction. Data are 3 independent replicates, except **b** (6 independent replicates) and **d** (4 independent replicates). Two-way ANOVA with Tukey’s post hoc test was performed for (**b**).

Excessive intracellular cystine accumulation may accelerate NADPH depletion, potentially inducing disulfidptosis [7]. However, cystine is converted intracellularly to cysteine, a key component of glutathione (GSH), which plays an essential antioxidant role [15, 16]. This suggests that cystine may have a dual role in cardiomyocytes (Fig. 2a). To explore the role of cystine in cardiomyocyte disulfidptosis, we used extracellular cystine deprivation. Compared with the OGD alone group, the OGD+ cystine deprivation group had elevated cell viability (Fig. 2b). At 3 and 6 h of OGD exposure, no significant reduction in intracellular cysteine levels was observed. This phenomenon may be attributed to glucose deprivation-induced inhibition of cysteine metabolism. However, a marked decrease in cysteine content was detected in the group subjected to combined OGD and cystine deprivation (Fig. 2d). Interestingly, cystine deprivation prevented NADPH depletion (Fig. 2c), and decreased OGD-induced disulfide bond formation in cytoskeletal proteins and alleviated morphological changes (Fig. 2d–f). These findings suggest that cells exhibit a certain degree of resistance to cysteine depletion during the initial phase of OGD. Although cystine deprivation leads to reduced cysteine levels, the concomitant decrease in NADPH consumption appears to mitigate cellular damage. We hypothesize that under OGD conditions, cystine deprivation primarily exerts its protective effects by preventing NADPH depletion, rather than through cysteine-related mechanisms.

### Dysregulation of the thioredoxin system mediates disulfidptosis in cardiomyocytes

**Fig. 3:**
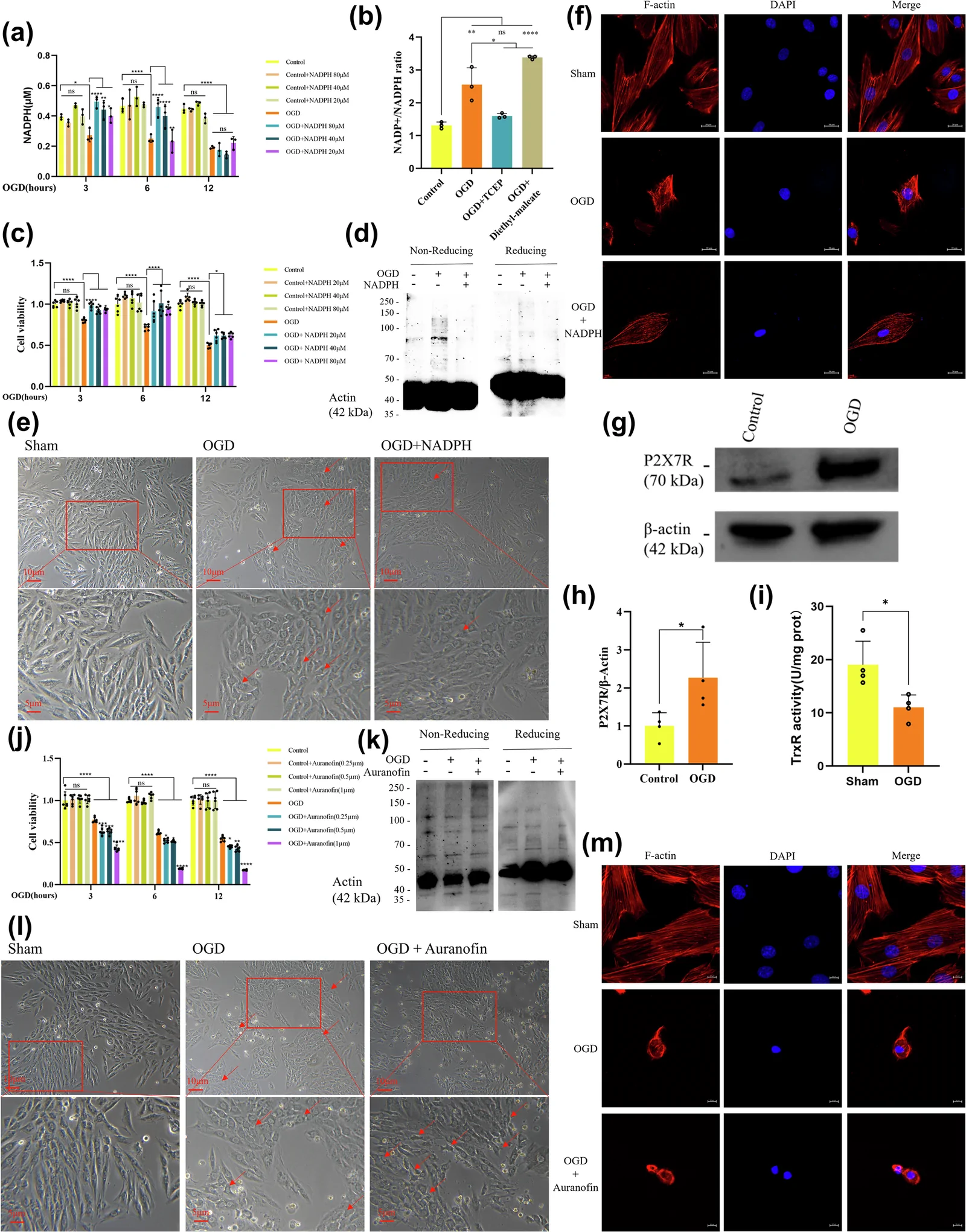
Dysregulation of the Trx system mediates disulfidptosis. **a** NADPH levels in H9C2 cells treated with Sham, Sham + NADPH (80 μM, 40 μM, 20 μM), OGD, and OGD + NADPH (80 μM, 40 μM, 20 μM) for 3, 6, and 12 h. **b** NADP+/NADPH ratio in H9C2 cells treated with Sham, OGD, TCEP (0.1 mM), and Diethylmaleate (0.3 mM) for 6 h. **c** CCK-8 assay of cell viability in H9C2 cells treated with Sham, Sham + NADPH (80 μM, 40 μM, 20 μM), OGD, and OGD + NADPH (80 μM, 40 μM, 20 μM) for 3, 6, and 12 h. **d** Western blot analysis of reduced and non-reduced Actin in H9C2 cells treated with Sham, OGD, and OGD + NADPH (80 μM) for 6 h. **e** Bright-field images of H9C2 cells treated with Sham, OGD, and OGD + NADPH (80 μM) for 6 h, captured at 200× magnification; red arrows indicate cellular morphology contraction. **f** Laser scanning confocal images showing F-actin fluorescence staining in H9C2 cells treated with Sham, OGD, and OGD + NADPH (80 μM) for 6 h. **g**, **h** Western blot analysis of P2X7R expression in Sham and OGD-treated H9C2 cells for 6 h. **g** Western blot images; **h** normalized P2X7R expression levels. **i** TrxR activity (U/mg protein) in Sham and OGD-treated H9C2 cells for 6 h. **j** CCK-8 assay of cell viability in H9C2 cells treated with Sham, Sham+Auranofin (1 μM, 0.5 μM, 0.25 μM), OGD, and OGD+Auranofin (1 μM, 0.5 μM, 0.25 μM) for 3, 6, and 12 h. **k** Western blot analysis of reduced and non-reduced Actin in H9C2 cells treated with Sham, OGD, and OGD + Auranofin (0.5 μM) for 6 h. **l** Bright-field images of H9C2 cells treated with Sham, OGD, and OGD+Auranofin (0.5 μM) for 6 h, captured at 200× magnification; red arrows indicate cellular contraction. **m** Laser scanning confocal images showing F-actin fluorescence staining in H9C2 cells treated with Sham, OGD, and OGD + Auranofin (0.5 μM) for 6 h. Data are 3 independent replicates, except **c**, **j** (6 independent replicates) and **g**, **h** (4 independent replicates). Two-way ANOVA with Tukey’s post hoc test was performed for (**a**, **c**, **j**), one-way ANOVA with Tukey’s post hoc test was performed for (**b**), unpaired Student’s *t*-test was performed in (**h**, **i**).

The Trx system comprises thioredoxin (Trx), thioredoxin reductase (TrxR), and NADPH [17]. Trx is a small (12 kDa), redox-active protein, while TrxR is an NADPH-dependent dimeric selenoenzyme containing an FAD domain. Reduced Trx functions as a highly efficient protein disulfide bond reductase, preventing excessive disulfide bond formation [18]. Oxidized Trx is restored to its reduced state by NADPH-dependent TrxR [17]. We next examined the role of NADPH in cardiomyocyte disulfidptosis. OGD treatment led to NADPH depletion in H9C2 cells. Interestingly, NADPH supplementation increased intracellular NADPH levels following OGD intervention but did not alter NADPH levels in the Sham group. NADPH supplementation was fully depleted by 12 h. (Fig. 3a) TCEP reduced the NADP+/NADPH ratio, while diethyl maleate increased it, indicating that disulfide bond formation plays a key role in NADPH depletion (Fig. 3b). Exogenous NADPH supplementation alleviated OGD-induced cellular damage and reduced disulfide bond formation in the cytoskeletal protein Actin (Fig. 3c, d). Additionally, NADPH mitigated changes in cell morphology and cytoskeletal protein collapse (Fig. 3e, f). Previous studies have suggested that NADPH may directly enter cells through activated P2X7R. Therefore, we examined the expression of P2X7R in cells and found that OGD (oxygen-glucose deprivation) induced a significant upregulation of P2X7R expression (Fig. 3g, h).

To further elucidate the mechanism of disulfidptosis in cardiomyocytes, we investigated the roles of additional components of the Trx system (TrxR). TrxR activity was significantly reduced in the OGD group (Fig. 3i). Treatment of H9C2 cells with Auranofin, a TrxR inhibitor, further exacerbated OGD-induced cellular damage (Fig. 3j). Additionally, Auranofin enhanced OGD-induced disulfide bond formation in cytoskeletal proteins (Fig. 3k), aggravating cellular deformation and cytoskeletal protein aggregation (Fig. 3l, m). These findings indicate that TrxR inactivation plays a key role in cardiomyocyte disulfidptosis.

### Exogenous NADPH supplementation attenuates myocardial infarction injury in mouse

**Fig. 4:**
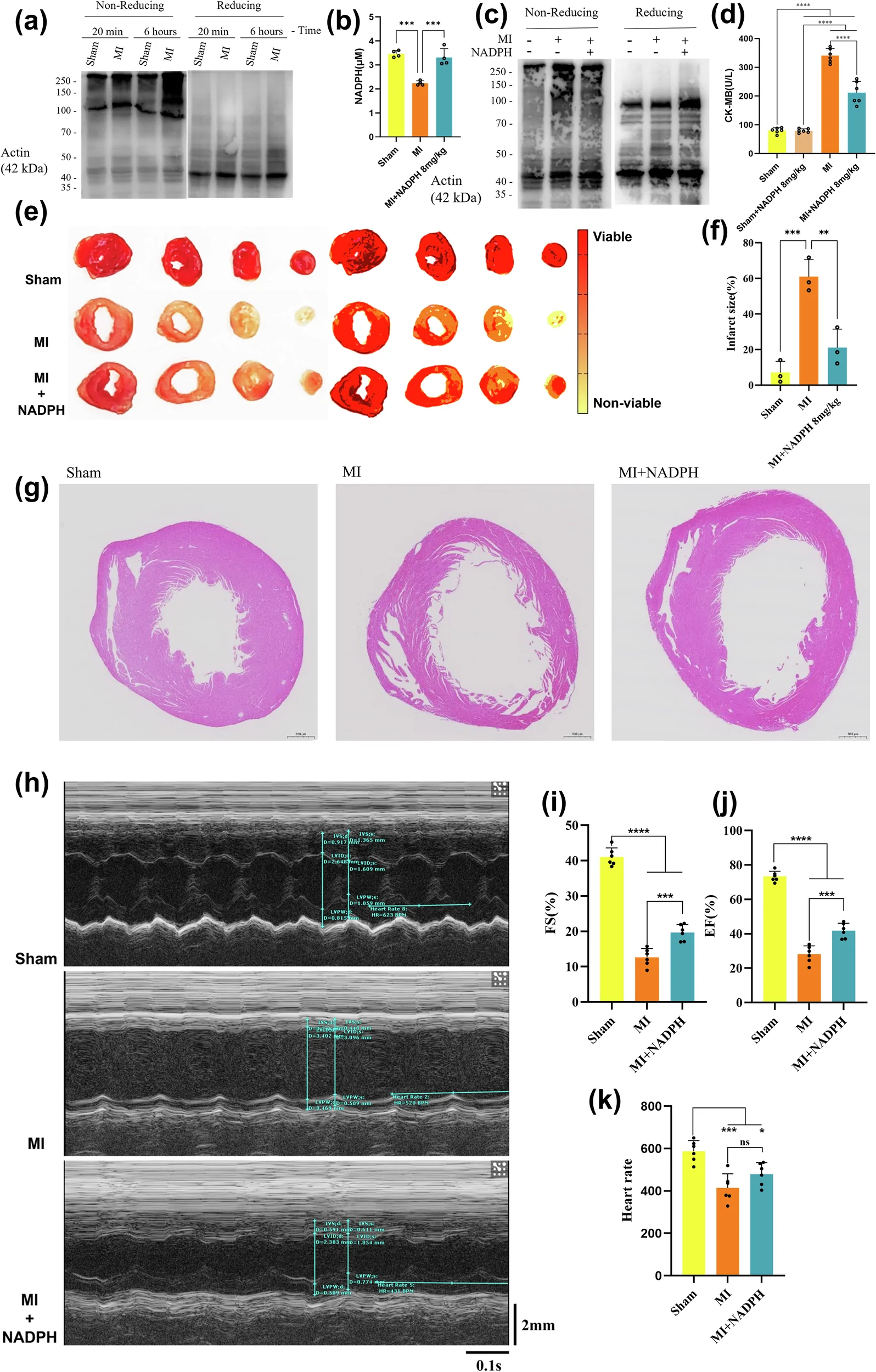
NADPH supplementation attenuates MI injury. **a** Western blot analysis of reduced and non-reduced Actin in cardiac tissues from Sham and MI groups at 20 min and 6 h. **b** NADPH levels in heart tissue of Sham, MI, and MI + NADPH (8 mg/kg) groups at 20 min. **c** Western blot analysis of reduced and non-reduced Actin in heart tissue from Sham, MI, and MI + NADPH (8 mg/kg) groups at 6 h. **e**, **f** TTC staining of hearts in Sham, MI, and MI + NADPH (8 mg/kg) groups at 6 h. **d** Serum CK-MB levels in Sham, Sham+NADPH (8 mg/kg), MI, and MI + NADPH (8 mg/kg) groups at 6 h. **e** TTC-stained heart sections; **f** percentage of non-viable myocardial area. **g** HE staining of hearts from Sham, MI, and MI + NADPH (8 mg/kg) groups at 6 h, shown at 2.5× magnification. **h**–**k** Echocardiographic assessment of cardiac function in Sham, MI, and MI + NADPH (8 mg/kg) groups at 6 h. **h** Echocardiographic M-mode images; **i** ejection fraction (EF, %); **j** fractional shortening (FS, %); **k** heart rate. Data are 3 independent replicates, except for (**d**, **h**–**k**) (6 independent replicates). One-way ANOVA with Tukey’s post hoc test was performed.

To verify the effectiveness of NADPH in vivo, we established a mouse model of myocardial infarction and intravenously administered 8 mg/kg NADPH via the tail vein immediately before surgery, referencing the dosage reported in previous studies [19, 20]. A time-dependent increase in disulfide bonds in cardiomyocyte skeletal proteins was observed, beginning in the early stages of myocardial injury (20 min) (Fig. 4a). Twenty min after surgery, NADPH levels at the myocardial injury site were significantly elevated in the MI + NADPH group (Fig. 4b). NADPH supplementation reduced disulfide bond formation in cardiomyocyte structural proteins (Fig. 4c). NADPH attenuated MI-induced ischemic injury in cardiomyocytes, as evidenced by TTC staining (Fig. 4e, f) and reduced serum CK-MB levels (a marker of myocardial injury) (Fig. 4d). HE staining revealed myocardial edema, congestion, necrosis, and inflammatory cell infiltration in the MI group, NADPH treatment significantly mitigated these pathological changes (Fig. 4g). Cardiac function was evaluated 6 h post-intervention, revealing a significant decline in EF% and FS% in the MI group, whereas NADPH administration markedly improved the MI-induced cardiac dysfunction (Fig. 4h–k).

### Proteomic analysis of myocardial cell

**Fig. 5:**
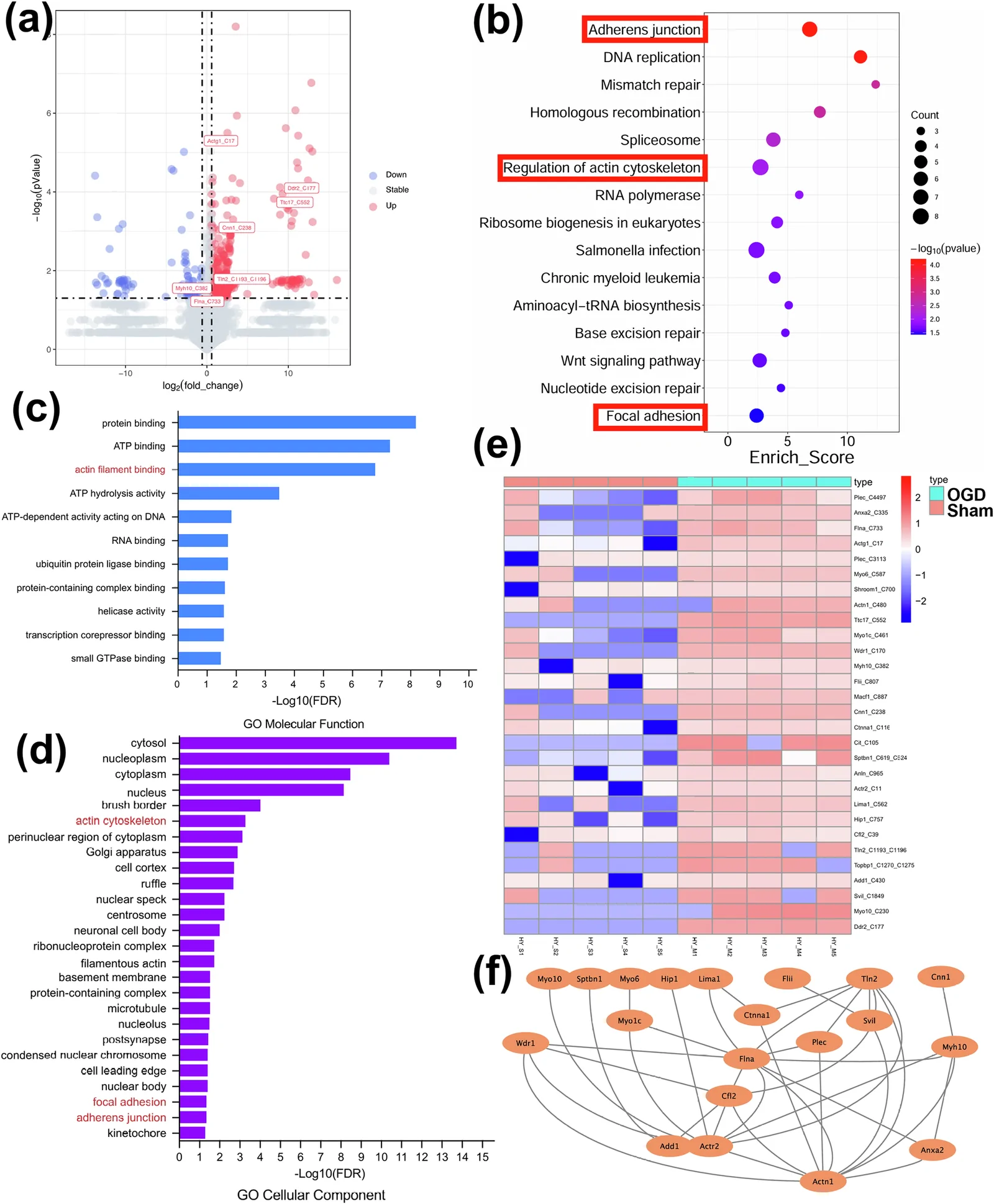
Proteomic analysis of myocardial cell. **a** Volcano plot. Red dots represent proteins exhibiting a ≥1.5-fold increase in detectable cysteine sites in the OGD group compared to the Sham group, while blue dots indicate proteins with a ≥1.5-fold decrease. **b** KEGG pathway enrichment analysis of proteins showing ≥2-fold increase in cysteine site detection. Pathways related to cytoskeletal organization are highlighted in red. **c**, **d** GO enrichment analysis of proteins with ≥1.5-fold increase in cysteine sites. Cytoskeleton-associated biological processes are marked in red. **e** Heatmap illustrating changes in cysteine site counts within actin cytoskeleton-related proteins. **f** Protein-protein interaction network of actin-associated proteins exhibiting ≥1.5-fold increase in cysteine site detection. Data are 5 independent replicates.

Disulfide bonds are formed through an oxidative reaction between the sulfhydryl groups (-SH) of two cysteine residues. Thus, the number of disulfide bonds is closely related to the number of cysteine sites [21]. Theoretically, the total number of cysteine residues within a given protein is invariant. However, the number of cysteine sites detectable by mass spectrometry can vary, likely due to conformational changes in proteins that affect enzymatic digestion efficiency. Specifically, a more open protein structure may expose more protease-accessible sites, including cysteine residues that are otherwise buried in stable structural regions. Interestingly, under oxygen-glucose deprivation (OGD) conditions, we observed a significantly larger number of proteins exhibiting a ≥1.5-fold increase in detectable cysteine sites compared to the Sham group, with 401 proteins up-regulated and 84 down-regulated (Fig. 5a). KEGG pathway analysis indicated that cell adhesion molecules and related cytoskeletal pathways were the most significantly enriched, with other cytoskeleton-associated pathways also showing notable enrichment (Fig. 5b). Gene Ontology (GO) analysis further confirmed that proteins with increased cysteine site counts were predominantly enriched in actin cytoskeleton-related biological processes (Fig. 5c, d). A heatmap visualization revealed up-regulation of cysteine site counts in actin cytoskeleton-related proteins following OGD treatment (Fig. 5e). Moreover, the protein-protein interaction network of actin-associated proteins showed a widespread increase of at least 1.5-fold after OGD (Fig. 5f).

These findings indicate that oxygen-glucose deprivation (OGD) leads to an increase in detectable cysteine sites within cytoskeleton-associated proteins. We hypothesize that this phenomenon may be closely related to alterations in protein conformation. The exposure of previously buried cysteine residues enhances their spatial accessibility, thereby facilitating disulfide bond formation under permissive conditions.

### Elevated calpain-1 expression promotes disulfidptosis in cardiomyocytes

**Fig. 6:**
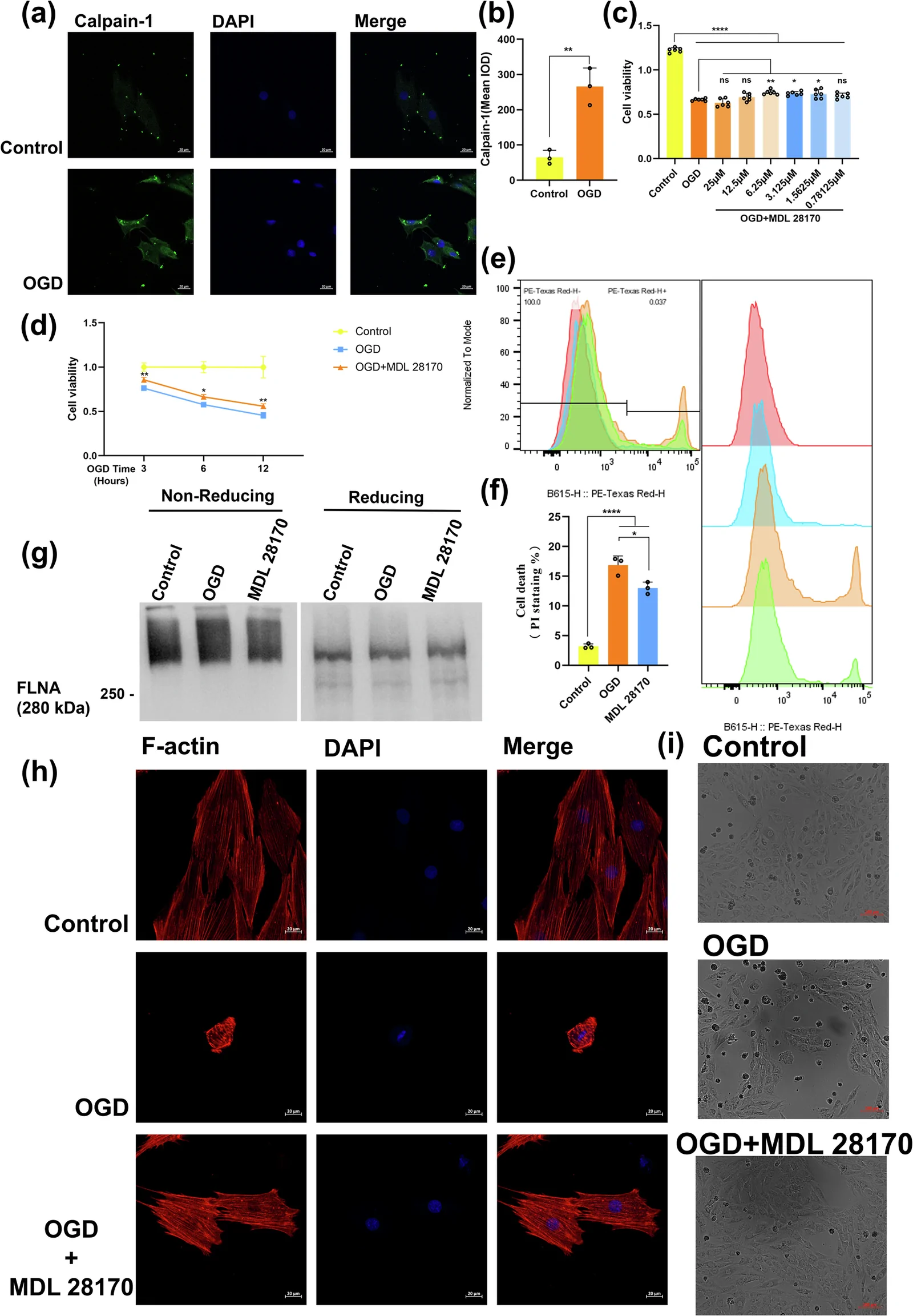
Elevated calpain-1 expression promotes disulfidptosis. **a** Immunofluorescence images of calpain-1 in Control group and OGD group (6 h). **b** Histogram of mean fluorescence intensity of calpain-1. **c** Cell viability measured by CCK-8 assay in Control group, OGD group (6 h), and OGD (6 h) + MDL 28170 (25 μM, 12.5 μM, 6.25 μM, 3.125 μM, 1.5625 μM, 0.78125 μM) groups. **d** Cell viability measured by CCK-8 assay in Control group, OGD group, and OGD + MDL 28170 (6.25 μM) group at 3, 6, and 12 h. Cell death detected by PI staining after 6 h in Control group, OGD group, and OGD + MDL 28170 (6.25 μM) group: **e** flow cytometry histograms, **f** percentage of PI-positive cells. **g** Western blot analysis of reduced and non-reduced FLNA in H9C2 cells treated with Control, OGD, and OGD + MDL 28170 (6.25 μM) for 6 h. **h** Laser scanning confocal microscopy images of H9C2 cells treated with Control, OGD, and OGD + MDL 28170 (6.25 μM) for 6 h. **i** Bright-field images of H9C2 cells treated with Control, OGD, and OGD + MDL 28170 (6.25 μM) for 6 h (200× magnification). Data are 3 independent replicates, except (**c**, **d**) (6 independent replicates). Two-way ANOVA with Tukey’s post hoc test was performed for (**d**), one-way ANOVA with Tukey’s post hoc test was performed for (**c**, **f**), unpaired Student’s *t*-test was performed in (**b**).

As substrates of calpain, cytoskeletal proteins can be hydrolyzed by this protease under pathological conditions [22], which may represent a key mechanism underlying conformational changes in cytoskeleton-associated proteins and create favorable conditions for disulfide bond formation. Our study demonstrated that OGD induces elevated expression of calpain-1 in H9C2 cells (Fig. 6a, b). Treatment with MDL 28170, a specific calpain-1 activity inhibitor, ameliorated the OGD-induced reduction in cell viability (Fig. 6c, d). Concurrently, MDL 28170 (6.25 μM) significantly attenuated OGD-induced cell death (Fig. 6e, f). Inhibition of calpain-1 also reduced disulfide bond formation in the cytoskeletal protein FLNA (Fig. 6g), alleviated cytoskeletal shrinkage (Fig. 6h), and improved overall cell morphology (Fig. 6i).

### Calcium overload-induced calpain activation promotes disulfidptosis

**Fig. 7:**
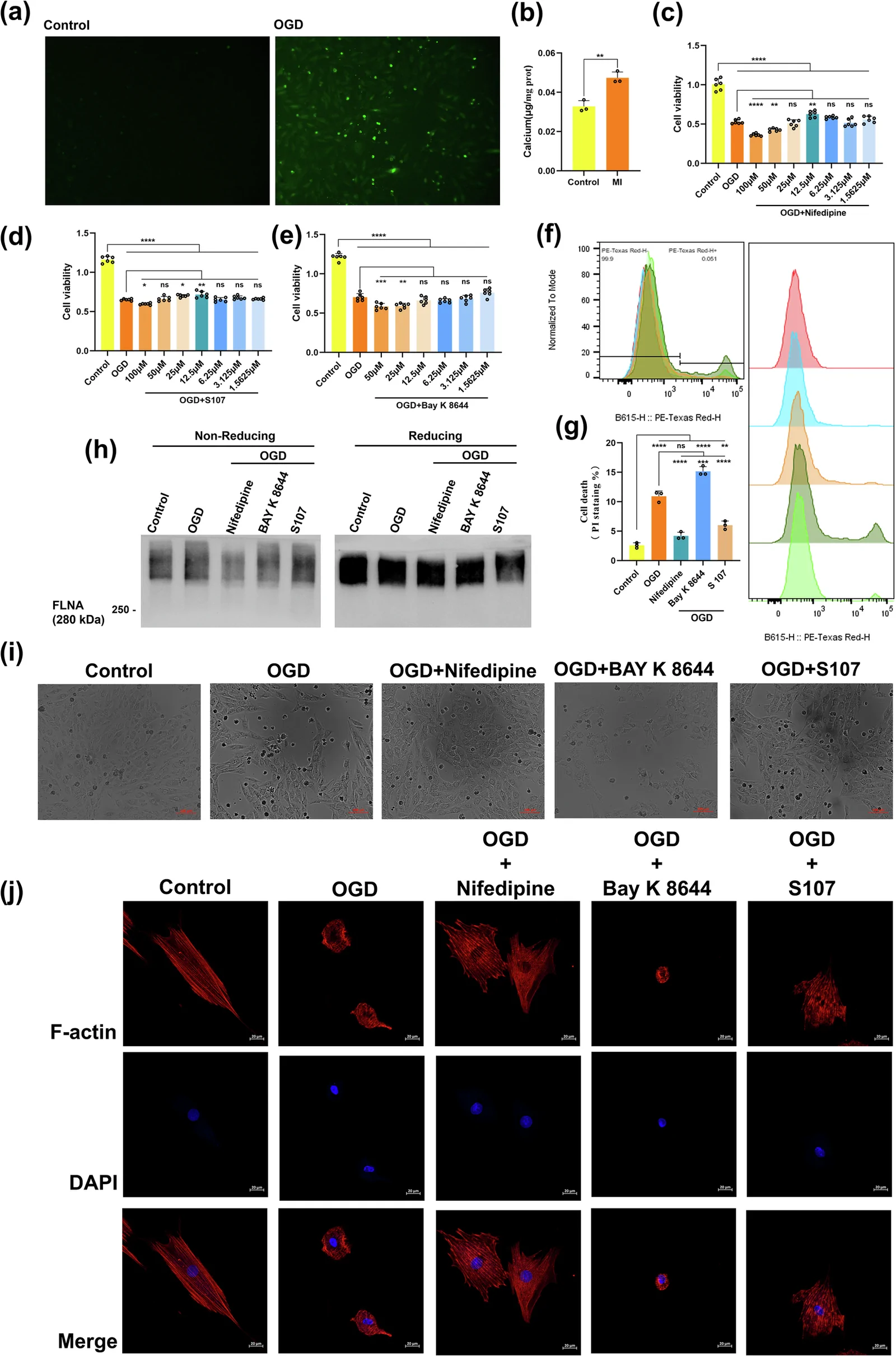
Calcium overload-induced calpain activation promotes disulfidptosis. **a** Free calcium levels detected by Fluo-4 AM fluorescence intensity in Control and OGD (6 h) groups. **b** Quantification of free calcium concentration using a calcium assay kit in Control and OGD (6 h) groups. **c** Cell viability measured by CCK-8 assay in Control, OGD (6 h), and OGD (6 h) + Nifedipine (1.56–100 μM) groups. **d** Cell viability in Control, OGD (6 h), and OGD (6 h) + S107 (1.56–100 μM) groups. **e** Cell viability in Control, OGD (6 h), and OGD (6 h) + Bay K 8644 (1.56–50 μM) groups. Cell death assessed by PI staining after 6 h: **f** flow cytometry histograms and **g** percentage of PI-positive cells in Control, OGD, Nifedipine (12.5 μM), OGD + S107 (12.5 μM), and OGD + Bay K 8644 (50 μM) groups. **h** Western blot analysis of FLNA under reducing and non-reducing conditions in H9C2 cells treated for 6 h with Control, OGD, Nifedipine (12.5 μM), OGD + S107 (12.5 μM), and OGD + Bay K 8644 (50 μM). **i** Bright-field images (200× magnification) of H9C2 cells under corresponding treatments for 6 h. **j** Laser scanning confocal images of cytoskeletal morphology in H9C2 cells after 6 h treatments. Data are 3 independent replicates, except (**c**, **d**, **e**) (6 independent replicates). One-way ANOVA with Tukey’s post hoc test was performed for (**c**, **d**, **e**, **g**), unpaired Student’s *t*-test was performed in (**b**).

As calcium-dependent proteases, calpains are known to be potently activated under conditions of calcium overload, as established in prior studies [23]. Initially, using calcium-sensitive fluorescent probes and direct quantification of intracellular calcium levels, we observed that oxygen-glucose deprivation (OGD) significantly increased the concentration of free calcium in H9C2 cells (Fig. 7a, b), which is expected to trigger aberrant calpain activation.

Treatment with nifedipine (an L-type calcium channel blocker) and S107 (a stabilizer of the ryanodine receptor RYR2 that inhibits calcium leak) attenuated the OGD-induced loss of cell viability (Fig. 7c, d), whereas Bay K 8644 (a calcium channel agonist) further reduced cell viability (Fig. 7e). Similarly, ameliorating calcium overload decreased OGD-induced cell death, while exacerbating calcium overload amplified cell death (Fig. 7f, g).

Notably, this calcium-associated regulation of cell death may be mediated through targeting disulfidptosis. Attenuating calcium overload reduced disulfide bond formation in the cytoskeletal protein FLNA, whereas promoting calcium accumulation enhanced disulfide cross-linking (Fig. 7h). Morphologically, these changes were accompanied by calcium overload-induced cytoskeletal aggregation and cellular shrinkage (Fig. 7I, j).

### Calpain mediates specific proteolysis of cytoskeletal proteins

**Fig. 8:**
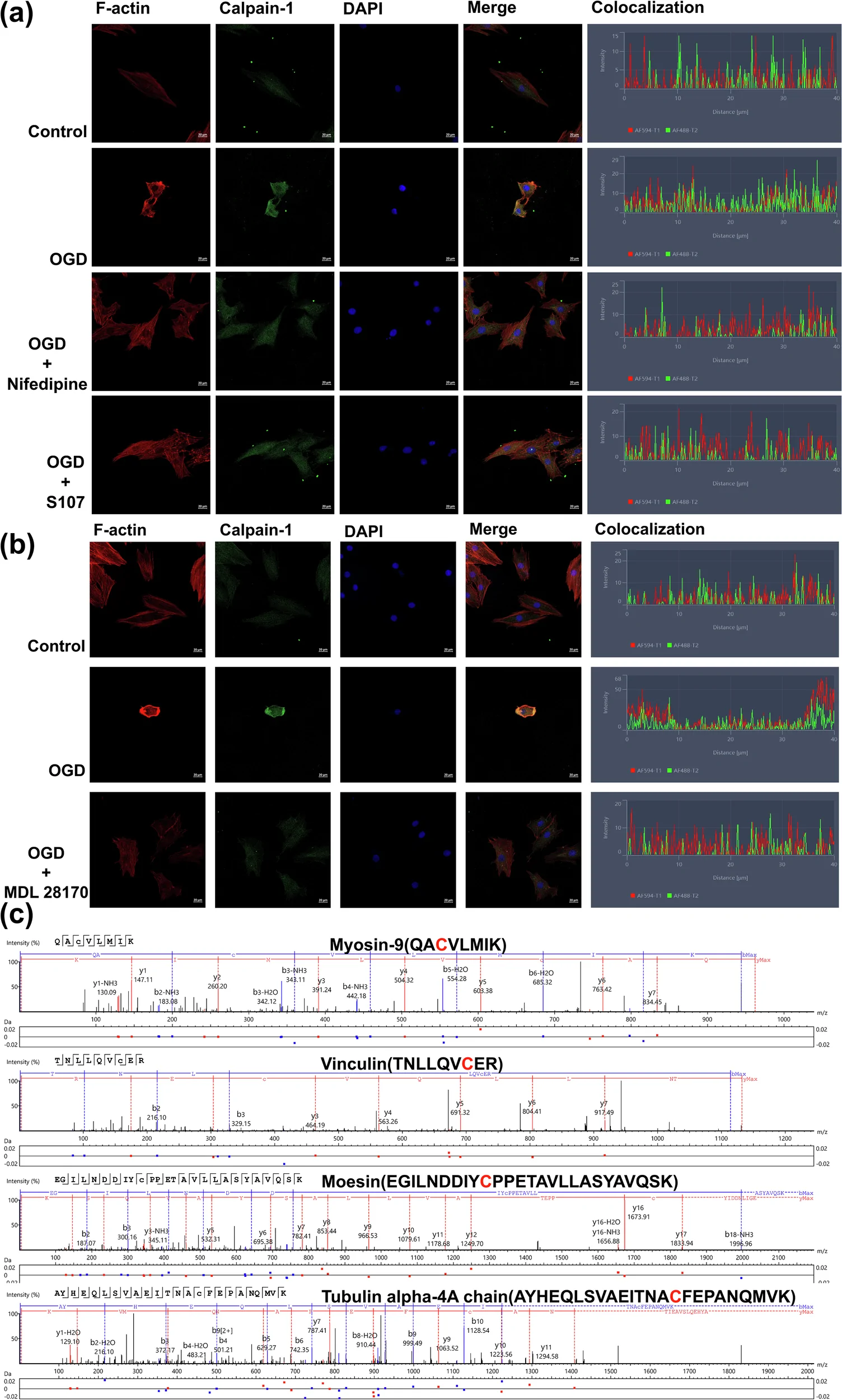
Calpain mediates specific proteolysis of cytoskeletal proteins. **a** Laser scanning confocal microscopy images and colocalization analysis of H9C2 cells treated for 6 h under Control, OGD, OGD + Nifedipine (12.5 μM), OGD + S107 (12.5 μM), and OGD + Bay K 8644 (50 μM) conditions. F-actin is shown in red, calpain-1 in green, and nuclei (DAPI) in blue. **b** Confocal images and colocalization analysis of H9C2 cells after 6-h treatments: Control, OGD, and OGD + MDL 28170 (6.25 μM). F-actin is labeled in red, calpain-1 in green, and nuclei (DAPI) in blue. **c** Cysteine residues and cysteine-containing peptides were analyzed by LC-MS/MS in Control, OGD (6 h), and OGD (6 h) + MDL 28170 (6.25 μM) groups, with the figure displaying peptides uniquely detected in the OGD group.

Our results demonstrated a significant increase in the colocalization of calpain with F-actin under OGD, suggesting that calpain may specifically target cytoskeletal proteins for proteolysis under OGD conditions. Treatment with either Nifedipine or S107, which alleviate calcium overload, markedly reduced the colocalization of calpain with F-actin (Fig. 8a). Similarly, direct inhibition of calpain using its specific inhibitor also suppressed their colocalization (Fig. 8b). These findings indicate that both suppression of calcium overload and direct inhibition of calpain activation can attenuate the interaction between calpain and cytoskeletal proteins.

To further investigate the functional consequences of calpain-mediated hydrolysis of cytoskeletal proteins, we performed protein Mass spectrometry and identified eight cysteine-containing peptides derived from cytoskeletal proteins—including Myosin-9, Vinculin, Unconventional myosin-Ic, Tubulin beta-5, Moesin, Tubulin alpha-1C chain, Tubulin alpha-4A chain, and Actin-related protein 2/3 complex subunit 1B—that were detected exclusively in the OGD group, but not in the Control or OGD + MDL 28170 groups (Fig. 8c; Supplementary Materials 1, Fig. 2).

This suggests that during OGD, calpain activation cleaves cytoskeletal proteins, thereby exposing cysteine residues that are typically buried within stable structural domains and making them more accessible for mass spectrometry detection. In contrast, inhibition of calpain prevents this proteolytic process, resulting in the absence of these cysteine-containing peptides in the mass spectrometry data. Ultimately, calpain-induced conformational changes and exposure of cysteine residues may provide the structural prerequisites for extensive disulfide bond formation under oxidative stress conditions.

### Calpain inhibition attenuates myocardial infarction-induced disulfidptosis

**Fig. 9:**
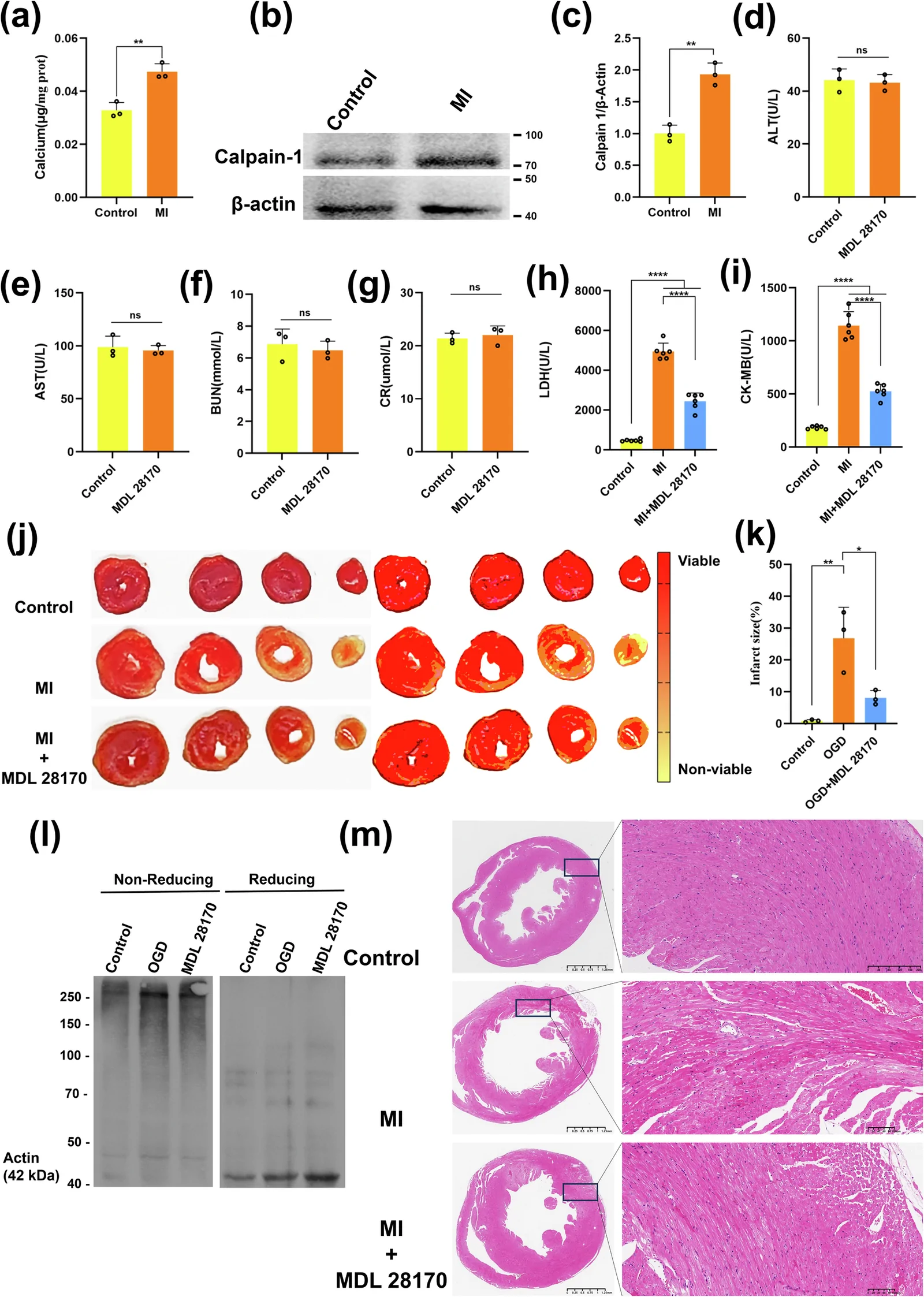
Calpain inhibition attenuates myocardial infarction-induced disulfidptosis. **a** Free calcium concentration in myocardial tissue from Control and MI groups at 6 h. Calpain-1 expression in myocardial tissue from Control and MI groups at 6 h: **b** representative Western blot images, **c** quantitative analysis of calpain-1 levels. **d**–**g** Serum levels of ALT, AST, BUN, and CR in Control and MDL 28170 (20 mg/kg/day, i.p., 3 days)-treated groups. **h**, **i** Serum CK-MB and LDH levels in Control, MI, and MI + MDL 28170 groups at 6 h. **j** TTC-stained heart sections. **k** percentage of non-viable myocardial area. **l** Western blot analysis of reduced and non-reduced FLNA in heart tissue from Sham, MI, and MI + MDL 28170 groups at 6 h. **m** HE staining of hearts from Sham, MI, and MI + MDL 28170 groups at 6 h. Data are 3 independent replicates, except **h**, **i** (6 independent replicates). One-way ANOVA with Tukey’s post hoc test was performed for (**h**, **i**, **k**), unpaired Student’s *t*-test was performed in (**a**, **c**, **d**, **e**, **f**, **g**).

Initial experiments confirmed the presence of calcium overload in myocardial tissue of the MI group (Fig. 9a), along with upregulated expression of calpain-1 (Fig. 9b, c). To investigate whether calpain activation contributes to MI-induced cardiac injury, mice were pretreated with MDL 28170 (20 mg/kg/day, intraperitoneally for 3 consecutive days) prior to MI induction [24, 25]. MDL 28170 pretreatment showed no adverse effects on liver or renal function (Fig. 9d–g), indicating a favorable safety profile.Treatment with MDL 28170 significantly reduced serum levels of myocardial injury markers (CK-MB and LDH; Fig. 9h, i), decreased myocardial infarct size (Fig. 9j, k), and alleviated histopathological damage (Fig. 9m). Echocardiographic results indicated that MDL28170 significantly improved cardiac function (Supplementary Materials 1, Fig. 2). Furthermore, MDL 28170 inhibited disulfide bond formation in FLNA (Fig. 9l), suggesting that its cardioprotective effects may be partially mediated through suppression of disulfidptosis.

## Discussion

This study confirms the occurrence of disulfidptosis in cardiomyocytes. Myocardial disulfidptosis can be triggered by oxygen-glucose deprivation (OGD) in vitro and is linked to myocardial ischemia and hypoxia in vivo. The underlying mechanism involves dysregulation of the Trx system, notably NADPH depletion and TrxR inactivation. Our study confirms that aberrant disulfide bond formation within the cytoskeletal proteins of cardiomyocytes leads to cellular contraction and cell death. This process is facilitated by both a pro-oxidant microenvironment resulting from Trx system dysfunction and structural alterations caused by calpain-mediated cleavage of cytoskeletal proteins.

Cellular damage caused by extensive disulfide bond formation in response to pathological stimuli is termed disulfide stress[26]. Disulfide stress arises when abnormal intermolecular disulfide bonds form between cysteine-rich proteins, with thiol groups undergoing irreversible oxidation to sulfinic and sulfonic acids [27, 28]. The concept of disulfidptosis suggests that, beyond disulfide stress due to NADPH depletion, cytoskeletal collapse occurs as disulfide bonds form within cytoskeletal proteins [7]. Our study confirmed the abnormal formation of disulfide bonds in cardiomyocyte structural proteins, resulting in cellular contraction and cell death. This study emphasizes the critical role of disulfidptosis in the early stages of myocardial infarction (MI). Accordingly, we selected 20 min and 6 h post-MI as endpoint time points. At 20 min, ischemia-induced NADPH depletion occurs, marking the initiation phase of disulfidptosis; thus, this time point is intended to capture the triggering event of disulfidptosis. The 6-hour time point is chosen to capture the execution phase of disulfidptosis, characterized by the physical collapse of the cytoskeleton and the establishment of irreversible damage.

MI creates a pathological state of myocardial hypoxia and a shortage of substrates needed for energy metabolism. Hypoxia in cardiomyocytes triggers an overproduction of reactive oxygen species (ROS), leading to oxidative stress and cellular injury [29, 30]. Oxidative stress is known to accelerate NADPH depletion [31]. Deficiencies in metabolic substrates (e.g., glucose) inhibit the pentose phosphate pathway (PPP), a major NADPH production pathway, leading to a marked reduction in NADPH levels [32]. NADPH is essential for the reduction of disulfide bonds. Our in vitro (OGD) and in vivo (MI) models showed a significant decrease in cardiomyocyte NADPH, which correlated with increased disulfide bond formation. Earlier studies suggested that NADPH cannot be acquired extracellularly and must be synthesized via intracellular metabolic pathways [33]. Recent studies, however, challenge this view, showing that exogenous NADPH can enter cells and perform its function [20, 34, 35]. The P2X7-R transporter may facilitate NADPH transport across the cell membrane [36, 37]. OGD induces upregulation of P2X7R expression. Exogenous NADPH supplementation significantly increased intracellular NADPH levels in the OGD group, but had no effect on NADPH levels in control cells. We speculate that this may be attributed to either OGD-induced activation of P2X7R, which allows NADPH entry into cells, or OGD-induced membrane rupture that leads to passive NADPH influx. Importantly, NADPH supplementation reduced both disulfide bond formation in cytoskeletal proteins and cell death in both in vivo (MI) and in vitro (OGD) models. However, we have only observed these phenomena, and the conclusion that NADPH directly enters cells still requires further experimental validation.

Cystine is transported into cells via the SLC7A11 transporter and requires substantial NADPH for its conversion to cysteine, contributing to disulfidptosis. Our study demonstrated that oxygen-glucose deprivation (OGD) significantly reduced intracellular NADPH levels, yet paradoxically maintained stable cysteine concentrations. This reservation may be associated with glucose deficiency-mediated suppression of cysteine catabolism [38], indicating cardiomyocytes exhibit temporal resistance to cysteine depletion during early OGD phases. While Slc7a11-mediated cystine uptake has been established as a critical regulator of ferroptosis in cardiomyocytes through GSH-mediated antioxidant mechanisms [39, 40], our findings revealed a paradoxical protective effect: cystine deprivation attenuated OGD-induced cellular injury by disulfidptosis. This apparent contradiction may be explained by the temporal dynamics of ferroptosis, which predominantly mediates late-stage cardiomyocyte death [41], whereas our experimental paradigm focused on acute injury phases. Notably, the beneficial effects of early cystine deprivation-particularly the conservation of NADPH reserves-appear to outweigh the detrimental consequences of GSH system impairment, ultimately conferring protection against acute cell death. Persistent inhibition of the Trx system disrupts intracellular redox homeostasis [42]. Our study demonstrates that OGD leads to a decrease in TrxR activity. Several studies have confirmed Trx inactivation due to nitrosylation during myocardial ischemia-reperfusion [43, 44]. Inhibition of TrxR activity led to an increase in disulfide bonds within cytoskeletal proteins.

Based on the susceptibility of cytoskeletal proteins to disulfide bond formation, we propose a novel concept of calpain-1-mediated “Cleavage–Fragmentation–Mismatch.” The activation of calpain-1 is primarily mediated through post-translational regulation and changes in the local microenvironment, rather than simply through increased gene transcription [45]. Our study demonstrates that ischemia disrupts myocardial calcium homeostasis, leading to the activation of calpain-1 and an increase in its expression due to calcium overload. This subsequently promotes the interaction between calpain-1 and cytoskeletal proteins, resulting in specific proteolytic cleavage of these proteins. Under stable protein conformations, spatial steric hindrance may prevent arbitrary disulfide bond formation between cysteine residues. Calpain-mediated cleavage disrupts the intact protein structure, thereby reducing steric constraints and increasing the likelihood of exposure of previously buried cysteine residues, which may subsequently form aberrant disulfide bonds. Concurrently, the pro-oxidizing intracellular environment induced by OGD further facilitates incorrect disulfide cross-linking among cysteine residues. Mass spectrometry analysis revealed a significant increase in detectable cysteine sites in the OGD group compared to both the control group and the OGD group treated with a calpain inhibitor.

This study is the first to report OGD-induced disulfidptosis in cardiomyocytes, highlighting the crucial roles of Trx system dysfunction and calpain-mediated hydrolysis of cytoskeletal proteins. Although direct research evidence is currently lacking, several potential peripheral blood biomarkers associated with disulfidptosis may reflect the severity of myocardial infarction in patients. These include NADPH levels and TrxR activity. Studies have confirmed that blood NADPH levels are decreased in patients with chronic heart failure [36]. TrxR, as a detectable protein in the blood, may exhibit altered activity or expression levels under various pathological conditions [46]. Therefore, it is reasonable to hypothesize that detecting these biomarkers in the peripheral blood of patients with myocardial infarction could provide insights into disease severity.

However, the research has several limitations: (1) The specific mechanism by which NADPH enters cells remains to be elucidated. (2) Potential crosstalk between disulfidptosis and other forms of cell death warrants further investigation. (3) The structural interpretations of cytoskeletal proteins in this study are based primarily on mass spectrometry and Western blotting; more direct techniques such as cryo-electron microscopy could be employed in future studies to better characterize protein structural changes. (4) There may be complex crosstalk between disulfidptosis and other forms of cell death, warranting further investigation in future studies to explore the interconnections among various cell death pathways. (5) Lack of genetic manipulation experiments (calpain-1 overexpression or knockdown/knockout).

## Methods and materials

### Reagents

SDS-PAGE Gel Quick Preparation Kit(Epizyme Biotech,PG111/PG112/PG113), Omni-ECL™ Enhanced Pico Light Chemiluminescence Kit(Epizyme Biotech,SQ101), Protein Sample Loading Buffer (Denaturing, non-Reducing) (Epizyme Biotech,LT103), Omni-Easy™Protein Sample Loading Buffer(Denaturing,Reducing) (Epizyme Biotech,LT101),Tris-Glycine-SDS(Epizyme Biotech,TF101),Tris-Glycine(Epizyme Biotech,TF102), Multicolor Prestained Protein Ladder(Epizyme Biotech,WJ103), Cell Counting Kit-8(MeilunBio,MA0218), Tris(2-carboxyethyl)phosphine hydrochloride (MedChemExpress,HY-W011500), Diethylmaleate(Yeasen, 53857ES60), Auranofin (MedChemExpress, HY-B1123)NADPH(Beyotime,ST360), Nifedipine(MCE, HY-B0284), S107 (MCE, HY-15292), Bay K 8644 (MCE, HY-10588), MDL-28170 (MCE, HY-18236),NADP+/NADPH Assay Kit with WST-8(Beyotime,S0179), Cysteine(Cys) Content Assay Kit(Solarbio, BC0180), Actin-Tracker Red-555(Beyotime, C2203S), Triton X-100(Beyotime, P0096), DAPI Staining Solution(Beyotime, C1005), PVDF(Millipore,0.45 µm, IPVH00010), HRP-conjugated Affinipure Goat Anti-Mouse IgG(H + L)(Proteintech, SA00001-1), Actin Monoclonal Antibody (ACTN05 (C4))(Thermo, MA5-11869), Thioredoxin Polyclonal antibody(Proteintech, 14999-1-AP), FLNA Monoclonal antibody(Proteintech, 67133-1-Ig), Alpha Tubulin Polyclonal antibody(Proteintech, 11224-1-AP), Calpain 1 Polyclonal antibody(Proteintech, 10538-1-AP), Multi-rAb™ CoraLite® Plus 488-Goat Anti-Rabbit Recombinant Secondary Antibody (H + L) (Proteintech, RGAR002), Beta Actin Monoclonal antibody(Proteintech, 66009-1-Ig)Propidium lodide(PI) (Yeasen, 40710ES03), Glucose-free DMEM(Pricella, PM150270), Glucose-and cystine-free DMEM(customized from Pricella), DMEM(Pricella,PM150210), Fetal Bovine Serum(Pricella, 164210),2,3, 5-triphenyltetrazolium chloride(TTC)(Sigma, T8877), Oxidized Thioredoxin Reductase(TrxR) Activity Assay Kit(Solarbio, BC1150), Fluo-4 AM(Beyotime, S1060), Calcium Colorimetric Assay Kit(Beyotime, S1063).

### Cell culture

Rat cardiomyocytes (H9C2) were purchased from Wuhan Pricella Biotechnology. The cell lines were verified by the vendor to be free of mycoplasma contamination. Cells were cultured in DMEM supplemented with 10% FBS and 1% penicillin/streptomycin, incubated at 37 °C with 5% CO₂. For OGD conditions, cells were maintained in glucose-free DMEM in a hypoxic environment (0.1% O₂).

### Mouse model of MI

Male C57BL/6J mice (20–25 g) were obtained from Shanghai Slaughter Laboratory Animal Co. All experimental procedures adhered to the Guide for the Care and Use of Laboratory Animals, established by the Ministry of Science and Technology of the People’s Republic of China. The animals were cared for in accordance with the Guidelines for Care and Use of Laboratory Animals published by the US National Institutes of Health (NIH Publication, 8th Edition, 2011). Sample size was based on the minimum number of animals required for robust histological assessment per ARRIVE guidelines. This study was approved by the Experimental Animal Ethics Committee of Shanghai University of Medicine & Health Sciences. Mouse were housed in specific-pathogen-free (SPF) grade facilities. The temperature was kept at 25 °C, with a 12-hour light-dark cycle, and mice were provided standard chow and water. Mice were divided into experimental groups via randomization. Animals were randomized using a computer-generated random number sequence. Investigators were blinded to group allocation during data acquisition and histological scoring. For the MI mouse model, anesthesia was performed using 2% isoflurane, and we adapted the myocardial infarction method described by Gao et al. [47], ligating the left anterior descending (LAD) branch of the coronary artery without ventilation. Whitening of the anterior wall of the left ventricle (LV) following ligation indicated successful model establishment. After 20 min/6 h, mice were euthanized via cervical dislocation.

### Cell Counting Kit-8(CCK-8)

Cell viability was assessed using the CCK-8 assay. Cells were seeded in 96-well plates at 5000 cells per well, 24 h prior to treatment. Following treatment, the medium was aspirated, wells were rinsed with PBS, and 100 µL of fresh medium with 10% CCK-8 reagent was added to each well. After a 2-hour incubation at 37 °C, absorbance was measured at 450 nm using a microplate reader. Cell viability (%) was calculated as: (absorbance of test well − absorbance of blank well)/(absorbance of control well − absorbance of blank well).

### Cell death assay

Cell mortality was quantified by the percentage of cells positively stained with propidium iodide (PI) [48]. Cells were seeded in 6-well plates at a density of 200,000 cells per well. Following treatment, cells were trypsinized, collected, and washed three times with ice-cold PBS, discarding the supernatant after each wash. Two milliliters of PI staining solution (500 nM) was added to each sample, which was then incubated in the dark for 15 min and kept on ice until analysis. The percentage of PI-positive cells was determined by flow cytometry (Agilent, NovoCyte).

### NADPH and NADP+/NADPH measurements

NADPH and NADP+/NADPH levels were measured following the kit instructions (Beyotime, S0179) [49]. Prior to intracellular NADPH quantification, cells were washed three times with phosphate-buffered saline (PBS). Prior to myocardial NADPH quantification, the heart was perfused and rinsed with physiological saline to remove residual blood components. Cell samples or heart tissue (ischemic region of the LV anterior wall) were collected and lysed with a specialized extraction solution. The supernatant was centrifuged and kept on ice for measurement. 10 μl of each sample were used for the BCA assay. For NADPH measurement, samples were heated to 60 °C for 30 min. Samples for the total NADP assay did not require heating. Fifty microliters of each sample were added to a 96-well plate, followed by 100 μL of G6PDH per well, and incubated at 37 °C for 10 min in the dark. Next, 10 μL of color development solution was added to each well, incubated at 37 °C for 60 min in the dark, and absorbance was measured at 450 nm using a microplate reader. The calculations were as follows: [NADP+] = [NADP_total] − [NADPH], and [NADP+]/[NADPH] = ([NADP_total] − [NADPH])/[NADPH]. NADPH values were normalized based on BCA assay data when represented independently.

### Western blot (WB)

Cells or tissues from the ischemic site in the LV anterior wall were collected, lysed with RIPA buffer, and centrifuged to collect supernatants. Protein concentrations were then measureed using the BCA protein assay kit. For reduced samples, denaturing, reduced-type loading buffer was added, followed by heating at 100 °C for 5 min. For non-reduced samples, denaturing, non-reducing loading buffer was added and heated to 60 °C for 10 min. Prepared samples were stored at −80 °C. Proteins were separated by electrophoresis on 7.5%, 10%, or 12.5% SDS-polyacrylamide gels, transferred to polyvinylidene fluoride membranes, and blocked for 20 min using a rapid blocking solution. Membranes were incubated overnight at 4 °C with primary antibodies (Actin, FLNA, Trx, Tubulin). After washing, membranes were incubated with secondary antibody at room temperature for 1 hour. Chemiluminescent signals were detected using an ECL solution. Protein bands were quantified and normalized using Image Lab software.

### Measurement of intracellular free calcium levels

The intracellular free calcium concentration was assessed using a Calcium Assay Kit (Beyotime, S1063) and the Fluo-4 AM fluorescent probe (Beyotime, S1060).

For the Calcium Assay Kit, measurements of calcium content in both cultured cells and myocardial tissues were performed strictly in accordance with the manufacturer’s instructions.

For Fluo-4 AM-based fluorescence detection, cells were first subjected to the designated treatments. After intervention, the culture medium was removed and cells were washed three times with PBS. Subsequently, Fluo-4 AM working solution (1 μM) was applied to the cells, followed by incubation at 37 °C for 30 min to allow probe loading. After incubation, cells were washed three times with PBS to remove excess probe. Images were acquired using an Olympus fluorescence inverted microscope under identical parameters (including laser intensity and exposure time) to ensure consistency.

### Bright-field imaging with microscope

Bright-field imaging was employed to assess cell growth density and morphology. Cells were seeded in 6-well plates as previously outlined and prepared for microscopic observation following treatment. Random bright-field images were captured at 200× magnification using Olympus microscope.

### Immunofluorescence staining

Cells were seeded in confocal dishes and subjected to the designated interventions. After treatment, the cells were washed twice with phosphate-buffered saline (PBS) and fixed with 4% paraformaldehyde for 30 min at room temperature. Following fixation, the cells were washed twice with PBS and permeabilized with 0.1% Triton X-100 for 10 min. After two additional PBS washes, the cells were blocked with 5% bovine serum albumin (BSA) for 1 h at room temperature and washed again twice with PBS.

The cells were then incubated overnight at 4 °C with a primary antibody against Calpain 1 (diluted 1:300 in PBS). After incubation, the cells were washed three times with PBS. Subsequently, they were incubated for 50 min at room temperature with a species-matched secondary antibody (diluted 1:500) and Actin-Tracker Red-594 (diluted 1:100) to label F-actin. The cells were then washed three times with PBS and incubated with DAPI (1 µg/mL) for 5 min to stain nuclei, followed by three final washes with PBS.

Images were acquired using a Leica confocal microscope. Colocalization analysis was performed with ZEN software (Carl Zeiss), and fluorescence intensity was quantified using Image-Pro Plus 6.0.

### Fluorescent staining of actin filaments

Actin fluorescent staining was employed to visualize the cell cytoskeleton [7]. Cells were seeded in confocal Petri dishes at 200,000 cells per well. After treatment, the medium was removed, and cells were washed three times with PBS. Cells were fixed with 4% paraformaldehyde for 15 min and permeabilized with Triton X-100 for 10 min. Cells were incubated with Actin-Tracker Red solution (5% BCA in PBS, 1:100 dilution) for 50 min at room temperature in the dark, followed by three PBS washes. DAPI was added for 5 min at room temperature, and cells were washed three times with PBS. Random fluorescence images were captured using a confocal microscope (LSM 880, Zeiss).

### TrxR activity assay

The TrxR activity assay was conducted following the manufacturer’s instructions [50]. Results were normalized based on BCA assay data.

### Cysteine detection

The cysteine assay was conducted following the manufacturer’s instructions. The results were normalized according to the BCA data of repeated holes.

### Proteomics

To extract proteins, cell samples were lysed using 8 M Urea and 100 mM Tris/HCl at a pH of 8.5. Protein concentrations were determined via the Bradford assay. An aliquot of the protein solution was then treated with 10 mM DTT and incubated at 56 °C with shaking at 600 rpm for 30 min. Add IAA to a final concentration of 50 mM and react for 30 min away from light. Add 4 fold volume of 50 mM Tris HCl (pH 8.0), dilute the UA concentration to 2 M. Trypsin was added at a protein: trypsin ratio of 50:1, and the sample was digested overnight (15–18 h) at 37 °C. To terminate the reaction, TFA was added to a final concentration of 0.1%, and the pH was adjusted to below 3 using 10% TFA. Finally, the peptides were purified using a C18 SPE cartridge and lyophilized for further analysis. Samples were processed using an EASY-nLC 1200 HPLC system set to a nanoliter flow rate. The separation employed two mobile phases: Mobile Phase A, consisting of 0.1% formic acid in water, and Mobile Phase B, a mixture of 0.1% formic acid with 80% acetonitrile. Initially, the column was equilibrated exclusively with Mobile Phase A. Enzymatic peptides were introduced to the pre-column (2 cm, ID100 μm, 3 μm, C18) via an autosampler and subsequently separated on a 15 cm analytical column (ID150 μm, 1.9 μm, C18) at a flow rate of 600 nL/min. The separated peptides were then analyzed using an OE480 mass spectrometer.

The detection mode is positive ion mode, the precursor ion scanning range is 300-1400 m/z, the resolution of primary mass spectrometry is 120,000 at 200 m/z, the AGC (Automatic gain control) target is 3e6, the Maximum IT is 80 ms, and the Dynamic exclusion time (Dynamic exclusion) is 40.0 s. The mass-to-charge ratios of peptides and peptide fragments were acquired as follows: each full scan was followed by a fragmentation profile (MS2 scan) with a total acquisition period of 1.5 s. The HCD fragmentation mode was used, with a Normalized Collision Energy of 30% and an Isolation window of 1.6 m/z. The resolution of the secondary MS was 7500 at 200 m/z. Qualitative and quantitative analysis of raw data from mass spectrometry using the iProteome one-stop data analysis cloud platform.

Samples were thawed on ice and lysed in RIPA buffer with 5 min of ultrasonication. After centrifugation at 12,000 × *g* and 4 °C for 15 min, the supernatant was collected and reduced with 10 mM DTT at 56 °C for 1 h, followed by alkylation with 20 mM IAA for 45 min in the dark.Protein concentration was determined using a Bradford assay with BSA standards (0–0.5 µg/µL). Samples and standards were loaded in triplicate into a 96-well plate, mixed with G250 dye, and absorbance was measured at 595 nm. For digestion, samples were diluted to 100 µL with 100 mM TEAB, trypsin was added, and incubation was carried out at 37 °C overnight. The digest was acidified with TFA, desalted using a C18 column, and lyophilized.The dried peptides were reconstituted in 0.1% formic acid and analyzed using a Vanquish NEO nanoLC system coupled to an Orbitrap Eclipse Tribrid mass spectrometer equipped with FAIMS. Separation was performed using a Thermo Scientific analytical column (25 cm × 75 μm, 2 μm) with a gradient of acetonitrile in 0.1% formic acid. MS data were acquired in DDA mode with FAIMS compensation voltages of –45 V and –65 V. Full MS scans (m/z 350–1500) were performed at 60,000 resolution, and MS/MS scans were triggered for the most intense ions with HCD fragmentation at 30% collision energy.Raw files were processed using Proteome Discoverer 3.1 with a 10 ppm precursor and 0.02 Da fragment mass tolerance. Identifications were filtered to include only PSMs and proteins with ≥ 99% confidence, at least one unique peptide, and an FDR < 1%.

### TTC Staining

TTC staining was used to evaluate myocardial viability and ischemic injury [51]. Six hours post-MI, mouse hearts were collected and washed twice with ice-cold PBS. Hearts were frozen at −80 °C for 15 min, then sliced into four sections of equal thickness from the ligature to the apex. Sections were incubated in 1% TTC solution at 37 °C for 10 min in the dark, and images were captured immediately afterward. Viable myocardium stained dark red, while non-viable tissue appeared white. The percentage of non-viable myocardial area was quantified using Image Pro Plus software.

### Hematoxylin and eosin (HE)

Mouse heart samples were collected 6 h post-MI. HE staining was conducted as previously described [52].

### Measurement of serum biochemical parameters

Serum samples were collected from mice and analyzed using a fully automated biochemical analyzer (XR210, XINRUI/LW C400) to determine the concentrations of the following biomarkers: creatine kinase-MB (CK-MB), lactate dehydrogenase (LDH), aspartate aminotransferase (AST), alanine aminotransferase (ALT), creatinine (CR), and blood urea nitrogen (BUN). All procedures were performed according to the manufacturer’s instructions.

### Echocardiography

Echocardiography was conducted on mouse 6 h post-myocardial infarction. Echocardiography was performed in B- and M-modes to measure left ventricular shortening fraction (LVFS) and ejection fraction (LVEF) using established formulae [53].

### Statistical analysis

Data are presented as mean ± standard deviation (SD). One-way ANOVA was applied for comparisons across multiple groups. For comparisons involving multiple groups with two independent variables, two-way ANOVA was used. An independent samples t-test was conducted for comparisons between two groups. Statistical significance is indicated as follows: *P < 0.05, **P < 0.01, ***P < 0.001, ****P < 0.0001.

## Supplementary information


Original Data
Supplementary Materials 1


## Data Availability

The datasets used and/or analysed during the current study are available from the corresponding author on reasonable request.

## References

[1] Martin SS, Aday AW, Allen NB, Almarzooq ZI, Anderson CAM, Arora P, et al. 2025 heart disease and stroke statistics: a report of US and global data from the American Heart Association. Circulation. 2025;151:e41–e660. 10.1161/CIR.0000000000001303. 39866113 PMC12256702

[2] Chong B, Jayabaskaran J, Jauhari SM, Chan SP, Goh R, Kueh MTW, et al. Global burden of cardiovascular diseases: projections from 2025 to 2050. Eur J Prev Cardiol. 2025;32:1001–15. 39270739 10.1093/eurjpc/zwae281

[3] Kaski JC, Crea F, Gersh BJ, Camici PG. Reappraisal of ischemic heart disease: fundamental role of coronary microvascular dysfunction in the pathogenesis of angina pectoris. Circulation. 2018;138:1463–80. 10.1161/CIRCULATIONAHA.118.031373. 30354347

[4] Heusch G. Myocardial ischemia/reperfusion: translational pathophysiology of ischemic heart disease. Med. 2024;5:10–31. 10.1016/j.medj.2023.12.007. 38218174

[5] Vince JE, Davidson NM, Tanzer MC. Necroptotic cell death consequences and disease relevance. Nat Immunol. 2025. 10.1038/s41590-025-02298-1. 41094200

[6] Chen X, Cai Q, Liang R, Zhang D, Liu X, Zhang M, et al. Copper homeostasis and copper-induced cell death in the pathogenesis of cardiovascular disease and therapeutic strategies. Cell Death Dis. 2023;14:105. 10.1038/s41419-023-05639-w. 36774340 PMC9922317

[7] Liu X, Nie L, Zhang Y, Yan Y, Wang C, Colic M, et al. Actin cytoskeleton vulnerability to disulfide stress mediates disulfidptosis. Nat Cell Biol. 2023;25:404–14. 10.1038/s41556-023-01091-2. 36747082 PMC10027392

[8] Jennings RB, Reimer KA. The cell biology of acute myocardial ischemia. Annu Rev Med. 1991;42:225–46. 10.1146/annurev.me.42.020191.001301. 2035969

[9] Geng W, Yan S, Li X, Liu Q, Zhang X, Gu X, et al. miR-432-5p Inhibits the Ferroptosis in Cardiomyocytes Induced by Hypoxia/Reoxygenation via Activating Nrf2/SLC7A11 Axis by Degrading Keap1. Anal Cell Pathol. 2023;2023:1293200. 10.1155/2023/1293200. PMC10564581 37822721

[10] Zhang Y, Ni Q, Chen Y, Pei Z, Xu X. The molecular mechanisms of disulfidptosis and its role in Diabetes mellitus and its complications. Biomed Pharmacother. 2025;191:118499. 10.1016/j.biopha.2025.118499. 40882526

[11] Bulaj G. Formation of disulfide bonds in proteins and peptides. Biotechnol Adv. 2005;23:87–92. 10.1016/j.biotechadv.2004.09.002. 15610970

[12] Azambuja F, Moons J, Parac-Vogt TN. The dawn of metal-oxo clusters as artificial proteases: from discovery to the present and beyond. Acc Chem Res. 2021;54:1673–84. 10.1021/acs.accounts.0c00666. 33600141

[13] Goll DE, Thompson VF, Li H, Wei W, Cong J. The calpain system. Physiol Rev. 2003;83:731–801. 10.1152/physrev.00029.2002. 12843408

[14] Huang Y, Wang KK. The calpain family and human disease. Trends Mol Med. 2001;7:355–62. 10.1016/s1471-4914(01)02049-4. 11516996

[15] Koppula P, Zhang Y, Zhuang L, Gan B. Amino acid transporter SLC7A11/xCT at the crossroads of regulating redox homeostasis and nutrient dependency of cancer. Cancer Commun. 2018;38:12. 10.1186/s40880-018-0288-x. PMC5993148 29764521

[16] Fujii J, Homma T, Kobayashi S. Ferroptosis caused by cysteine insufficiency and oxidative insult. Free Radic Res. 2020;54:969–80. 10.1080/10715762.2019.1666983. 31505959

[17] Holmgren A. Thioredoxin. Annu Rev Biochem. 1985;54:237–71. 10.1146/annurev.bi.54.070185.001321. 3896121

[18] Lillig CH, Holmgren A. Thioredoxin and related molecules-from biology to health and disease. Antioxid Redox Signal. 2007;9:25–47. 10.1089/ars.2007.9.25. 17115886

[19] Qin YY, Li M, Feng X, Wang J, Cao L, Shen XK, et al. Combined NADPH and the NOX inhibitor apocynin provides greater anti-inflammatory and neuroprotective effects in a mouse model of stroke. Free Radic Biol Med. 2017;104:333–45. 10.1016/j.freeradbiomed.2017.01.034. 28132925

[20] Zhou JS, Zhu Z, Wu F, Zhou Y, Sheng R, Wu JC, et al. NADPH ameliorates MPTP-induced dopaminergic neurodegeneration through inhibiting p38MAPK activation. Acta Pharm Sin. 2019;40:180–91. 10.1038/s41401-018-0003-0. PMC6329815 29769744

[21] Kiss A, Elhawat N, Kovács Z, Kaszás L, Béni Á, Domokos-Szabolcsy É, et al. Optimizing mung bean and soybean hydrolysis for the generation of bioactive peptides of potential functional food applications. Food Chem: X. 2025;30:102925. 41049777 10.1016/j.fochx.2025.102925 PMC12491734

[22] Ma M. Role of calpains in the injury-induced dysfunction and degeneration of the mammalian axon. Neurobiol Dis. 2013;60:61–79. 10.1016/j.nbd.2013.08.010. 23969238 PMC3882011

[23] Inserte J, Hernando V, Garcia-Dorado D. Contribution of calpains to myocardial ischaemia/reperfusion injury. Cardiovasc Res. 2012;96:23–31. 10.1093/cvr/cvs232. 22787134

[24] Wei S, Liu Y, Ran C, Li Y, Tang B, Lu M, et al. Calpain-1 up-regulation promotes Bleomycin-Induced pulmonary fibrosis by activating ferroptosis. Am J Pathol. 2024;194:2272–89. 10.1016/j.ajpath.2024.09.004. 39326733

[25] Liu L, Wang Y, Wang X, Zhang G, Sha S, Zhou R, et al. Transient receptor potential vanilloid 4 blockage attenuates pyroptosis in hippocampus of mice following pilocarpine‑induced status epilepticus. Acta Neuropathol Commun. 2025;13:73. 10.1186/s40478-025-01990-5. 40205503 PMC11983898

[26] Moreno ML, Escobar J, Izquierdo-Álvarez A, Gil A, Pérez S, Pereda J, et al. Disulfide stress: a novel type of oxidative stress in acute pancreatitis. Free Radic Biol Med. 2014;70:265–77. 10.1016/j.freeradbiomed.2014.01.009. 24456905

[27] Qian S, Chen G, Li R, Ma Y, Pan L, Wang X, et al. Disulfide stress and its role in cardiovascular diseases. Redox Biol. 2024;75:103297. 10.1016/j.redox.2024.103297. 39127015 PMC11364009

[28] Chauvin JR, Pratt DA. On the reactions of thiols, sulfenic acids, and sulfinic acids with hydrogen peroxide. Angew Chem Int Ed Engl. 2017;56:6255–59. 10.1002/anie.201610402. 27933690

[29] Wang K, Yao SY, Wang Z, Shen L, Guo DS, Zhu Y, et al. A sequential dual functional supramolecular hydrogel with promoted drug release to scavenge ROS and stabilize HIF-1α for myocardial infarction treatment. Adv Health Mater. 2024;13:e2302940. 10.1002/adhm.202302940. 37844263

[30] Qi B, Song L, Hu L, Guo D, Ren G, Peng T, et al. Cardiac-specific overexpression of Ndufs1 ameliorates cardiac dysfunction after myocardial infarction by alleviating mitochondrial dysfunction and apoptosis. Exp Mol Med. 2022;54:946–960. 10.1038/s12276-022-00800-5. PMC9355970 35817848

[31] Fernandez-Marcos PJ, Nóbrega-Pereira S. NADPH: new oxygen for the ROS theory of aging. Oncotarget. 2016;7:50814–15. 10.18632/oncotarget.10744. 27449104 PMC5239434

[32] TeSlaa T, Ralser M, Fan J, Rabinowitz JD. The pentose phosphate pathway in health and disease. Nat Metab. 2023;5:1275–89. 10.1038/s42255-023-00863-2. 37612403 PMC11251397

[33] Liu X, Zhang Y, Zhuang L, Olszewski K, Gan B. NADPH debt drives redox bankruptcy: SLC7A11/xCT-mediated cystine uptake as a double-edged sword in cellular redox regulation. Genes Dis. 2020;8:731–45. 10.1016/j.gendis.2020.11.010. 34522704 PMC8427322

[34] Zhu J, Wang YF, Chai XM, Qian K, Zhang LW, Peng P, et al. Exogenous NADPH ameliorates myocardial ischemia-reperfusion injury in rats through activating AMPK/mTOR pathway. Acta Pharm Sin. 2020;41:535–45. 10.1038/s41401-019-0301-1. PMC7470878 31776448

[35] Liu N, Wu WL, Wan XR, Wang J, Huang JN, Jiang YY, et al. Regulation of FSP1 myristoylation by NADPH: a novel mechanism for ferroptosis inhibition. Redox Biol. 2024;73:103176. 10.1016/j.redox.2024.103176. 38705094 PMC11074979

[36] Qian K, Tang J, Ling YJ, Zhou M, Yan XX, Xie Y, et al. Exogenous NADPH exerts a positive inotropic effect and enhances energy metabolism via SIRT3 in pathological cardiac hypertrophy and heart failure. EBioMedicine. 2023;98:104863. 10.1016/j.ebiom.2023.104863. 37950995 PMC10663691

[37] Mou YJ, Li FM, Zhang R, Sheng R, Han R, Zhang ZL, et al. The P2X7 receptor mediates NADPH transport across the plasma membrane. Biochem Biophys Res Commun. 2024;737:150500. 10.1016/j.bbrc.2024.150500. 39142135

[38] Bak DW, Bechtel TJ, Falco JA, Weerapana E. Cysteine reactivity across the subcellular universe. Curr Opin Chem Biol. 2019;48:96–105. 10.1016/j.cbpa.2018.11.002. 30508703 PMC6382561

[39] Fang X, Cai Z, Wang H, Han D, Cheng Q, Zhang P, et al. Loss of cardiac ferritin H facilitates cardiomyopathy via Slc7a11-mediated ferroptosis. Circ Res. 2020;127:486–501. 10.1161/CIRCRESAHA.120.316509. 32349646

[40] Ichihara G, Katsumata Y, Sugiura Y, Matsuoka Y, Maeda R, Endo J, et al. MRP1-dependent extracellular release of glutathione induces cardiomyocyte ferroptosis after ischemia-reperfusion. Circ Res. 2023;133:861–76. 10.1161/CIRCRESAHA.123.323517. 37818671

[41] Cai W, Liu L, Shi X, Liu Y, Wang J, Fang X, et al. Alox15/15-HpETE aggravates myocardial ischemia-reperfusion injury by promoting cardiomyocyte ferroptosis. Circulation. 2023;147:1444–60. 10.1161/CIRCULATIONAHA.122.060257. 36987924

[42] Holmgren A. Thioredoxin and glutaredoxin systems. J Biol Chem. 1989;264:13963–6. 2668278

[43] Tao L, Jiao X, Gao E, Lau WB, Yuan Y, Lopez B, et al. Nitrative inactivation of thioredoxin-1 and its role in postischemic myocardial apoptosis. Circulation. 2006;114:1395–402. 10.1161/CIRCULATIONAHA.106.625061. 16966583

[44] Zhang H, Tao L, Jiao X, Gao E, Lopez BL, Christopher TA, et al. Nitrative thioredoxin inactivation as a cause of enhanced myocardial ischemia/reperfusion injury in the aging heart. Free Radic Biol Med. 2007;43:39–47. 10.1016/j.freeradbiomed.2007.03.016. 17561092 PMC1949486

[45] Zhang X, Zheng Y, Wang Z, Zhang G, Yang L, Gan J, et al. Calpain: the regulatory point of cardiovascular and cerebrovascular diseases. Biomed Pharmacother. 2024;179:117272. 10.1016/j.biopha.2024.117272. 39153432

[46] Branco V, Carvalho L, Barboza C, Mendes E, Cavaco A, Carvalho C. Selenium and redox enzyme activity in pregnant women exposed to methylmercury. Antioxid. 2022;11:2291. 10.3390/antiox11112291. PMC9687717 36421477

[47] Gao E, Lei YH, Shang X, Huang ZM, Zuo L, Boucher M, et al. A novel and efficient model of coronary artery ligation and myocardial infarction in the mouse. Circ Res. 2010;107:1445–53. 10.1161/CIRCRESAHA. 20966393 PMC3005817

[48] Tang X, Guo M, Ding P, Deng Z, Ke M, Yuan Y, et al. BUB1B and circBUB1B_544aa aggravate multiple myeloma malignancy through evoking chromosomal instability. Signal Transduct Target Ther. 2021;6:361. 34620840 10.1038/s41392-021-00746-6 PMC8497505

[49] Yin X, Peng J, Gu L, Liu Y, Li X, Wu J, et al. Targeting glutamine metabolism in hepatic stellate cells alleviates liver fibrosis. Cell Death Dis. 2022;13:955. 36376267 10.1038/s41419-022-05409-0 PMC9663710

[50] Yu W, Chen C, Zhuang W, Wang W, Liu W, Zhao H, et al. Silencing TXNIP ameliorates high uric acid-induced insulin resistance via the IRS2/AKT and Nrf2/HO-1 pathways in macrophages. Free Radic Biol Med. 2022;178:42–53. 10.1016/j.freeradbiomed. 34848368

[51] Hu J, Li Z, Xu LT, Sun AJ, Fu XY, Zhang L, et al. Protective effect of apigenin on ischemia/reperfusion injury of the isolated rat heart. Cardiovasc Toxicol. 2015;15:241–9. 10.1007/s12012-014-9290-y. 25377428

[52] Qin Z, Wang X, Zhou Y, Zheng J, Li H, Li L. Upregulation of miR-29b-3p alleviates coronary microembolization-induced myocardial injury via regulating BMF and GSK-3β. Apoptosis. 2023;28:210–21. 10.1007/s10495-022-01788-z. 36315357

[53] Kovilakath A, Mauro AG, Valentine YA, Raucci FJ, Jamil M, Carter C, et al. SPTLC3 is essential for complex I activity and contributes to ischemic cardiomyopathy. Circulation. 2024;150:622–41. 10.1161/CIRCULATIONAHA.123.066879. 38660786 PMC11333184

