# Supplementary material for: Thioredoxin system dysregulation and calpain activation drive myocardial disulfidptosis via pathological disulfide bonds remodeling
Source: Cell Death Discov. 2026 Jun 19;12:350. doi: 10.1038/s41420-026-03200-0 (PMC13521956; doi:10.1038/s41420-026-03200-0)

For exposure pictures where the PVDF membrane boundary could not be seen clearly, we additionally supplemented the PVDF membrane photo taken by the camera.

Fig.1b

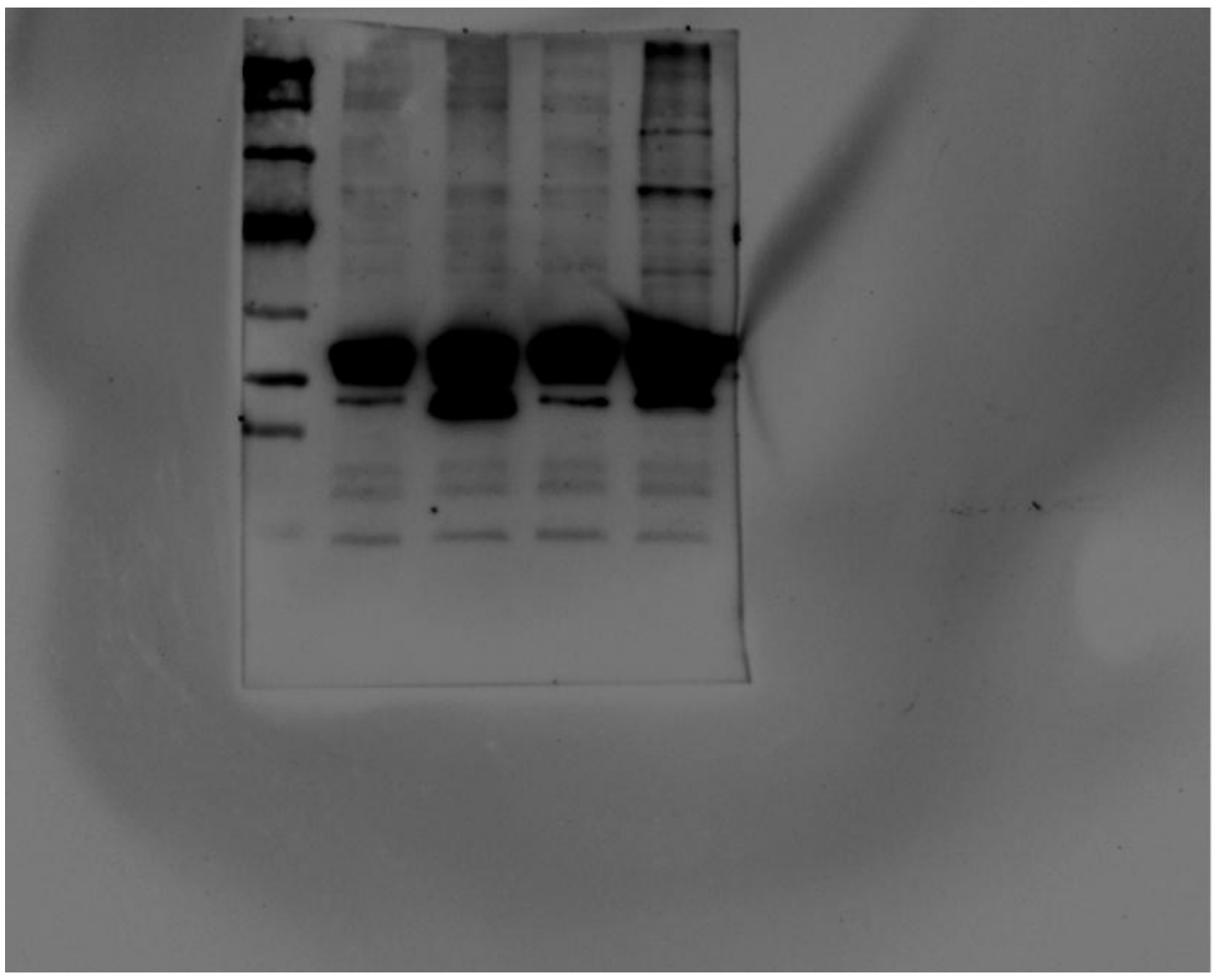

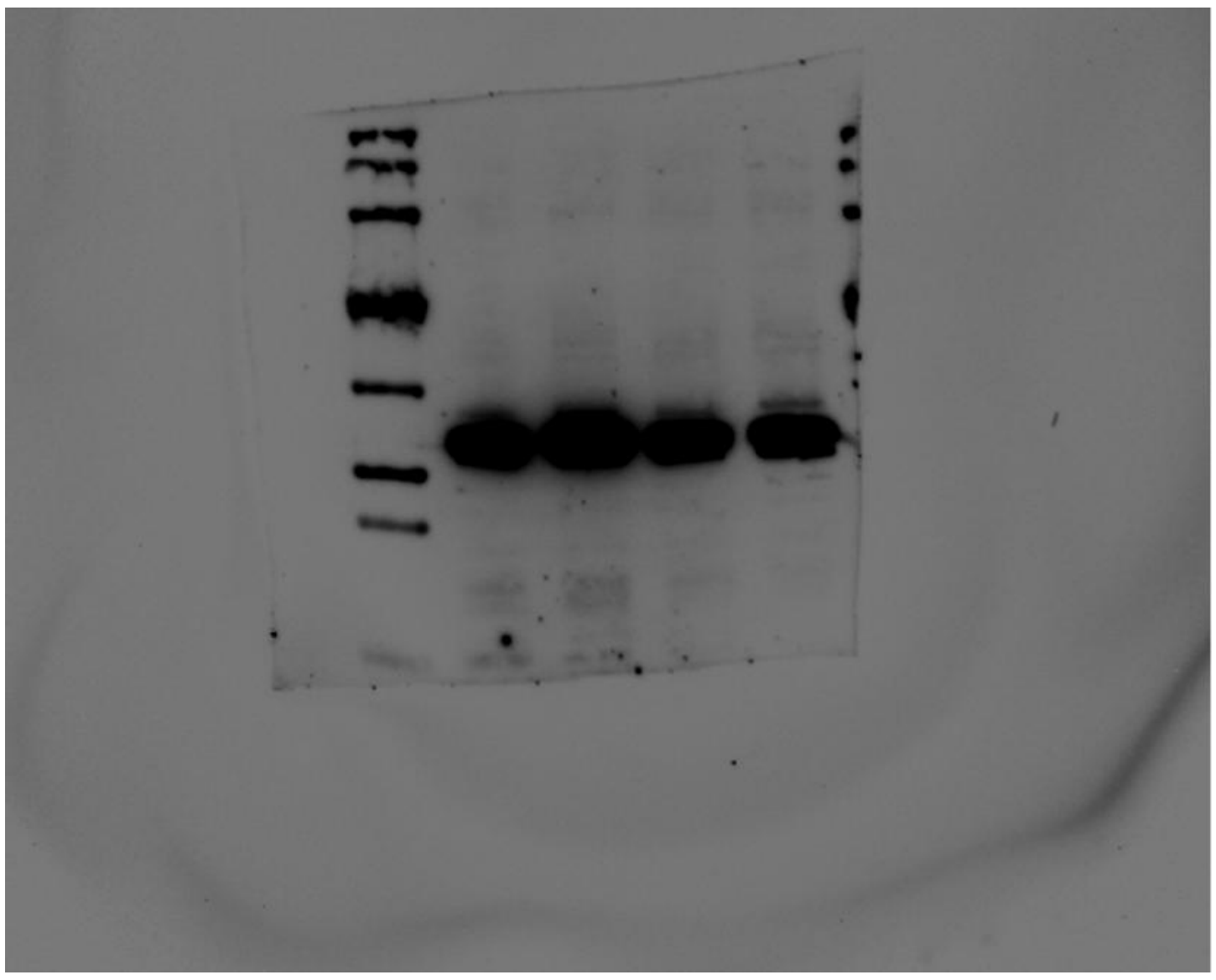

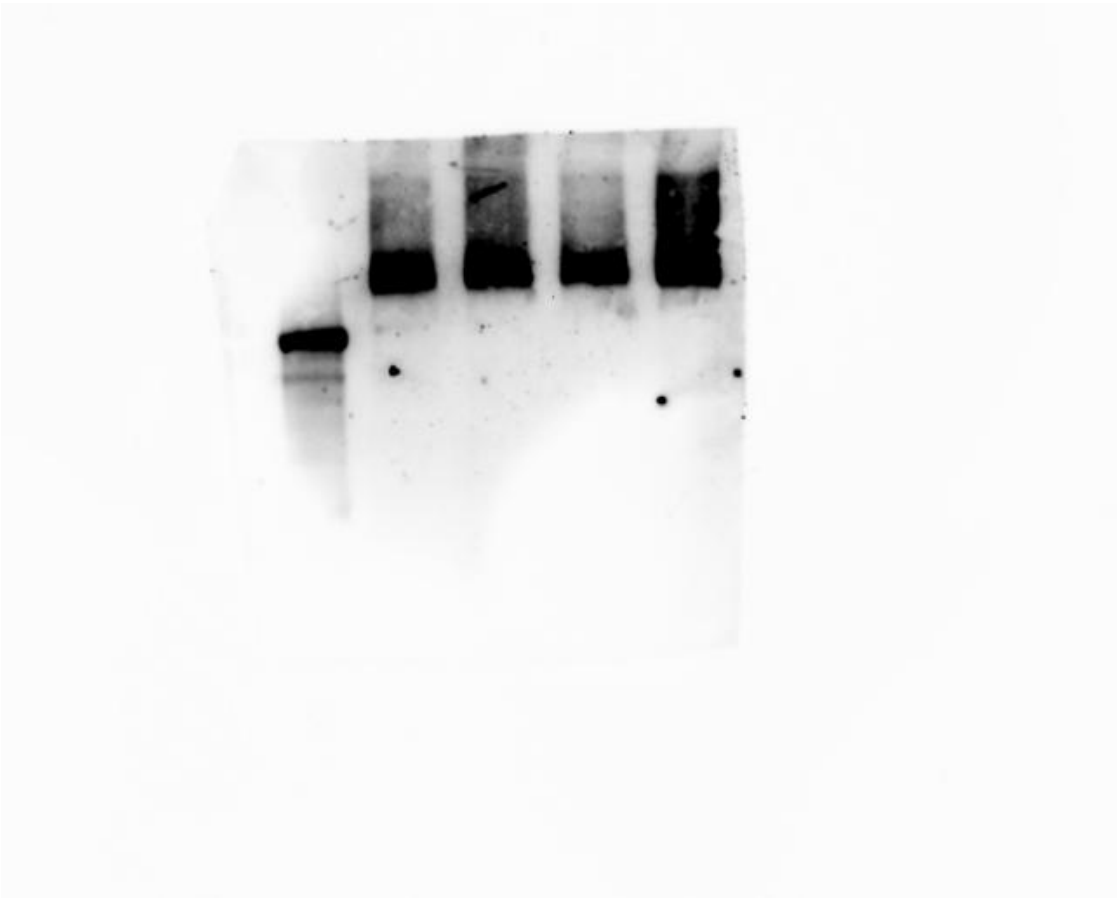

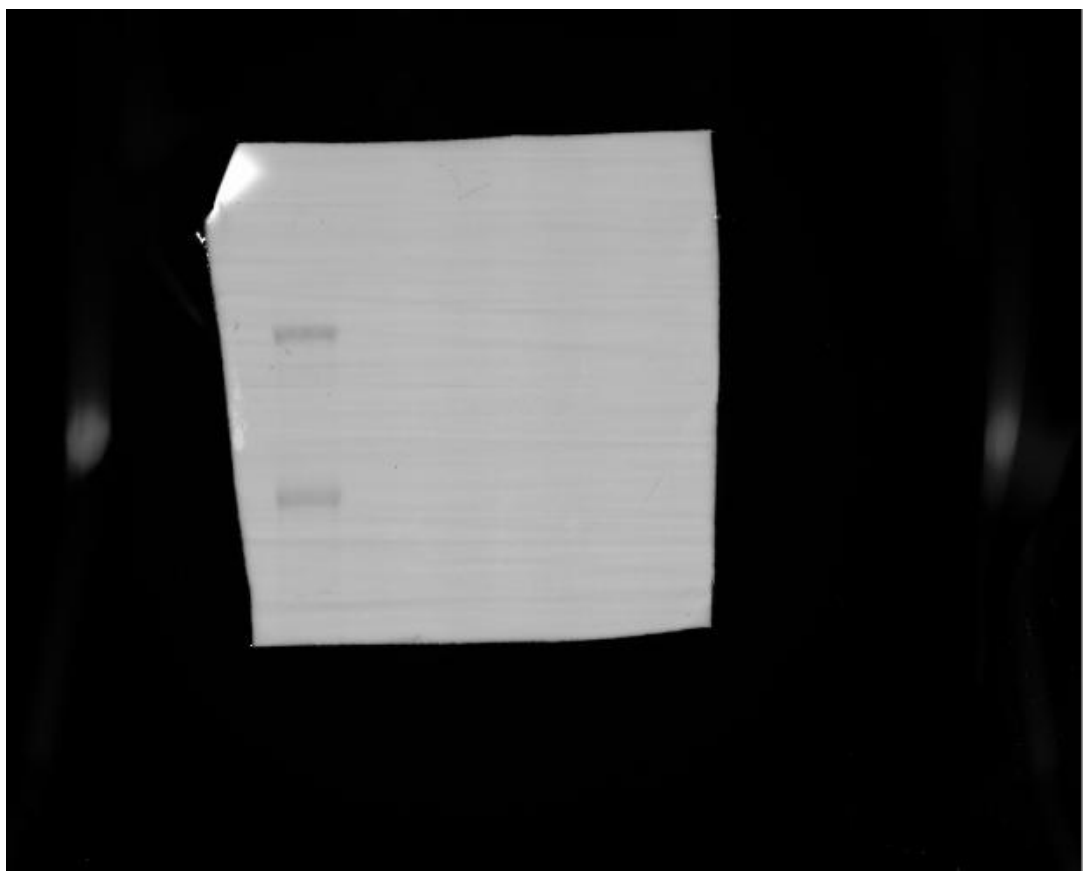

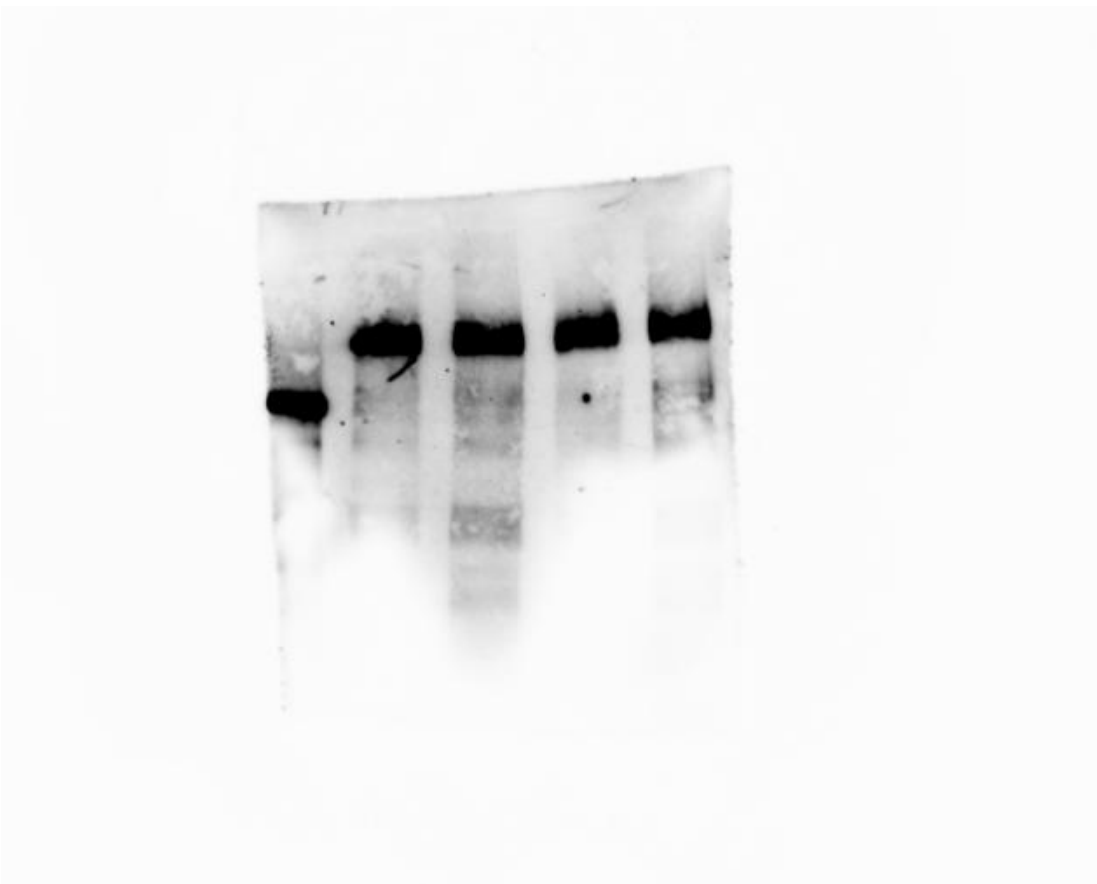

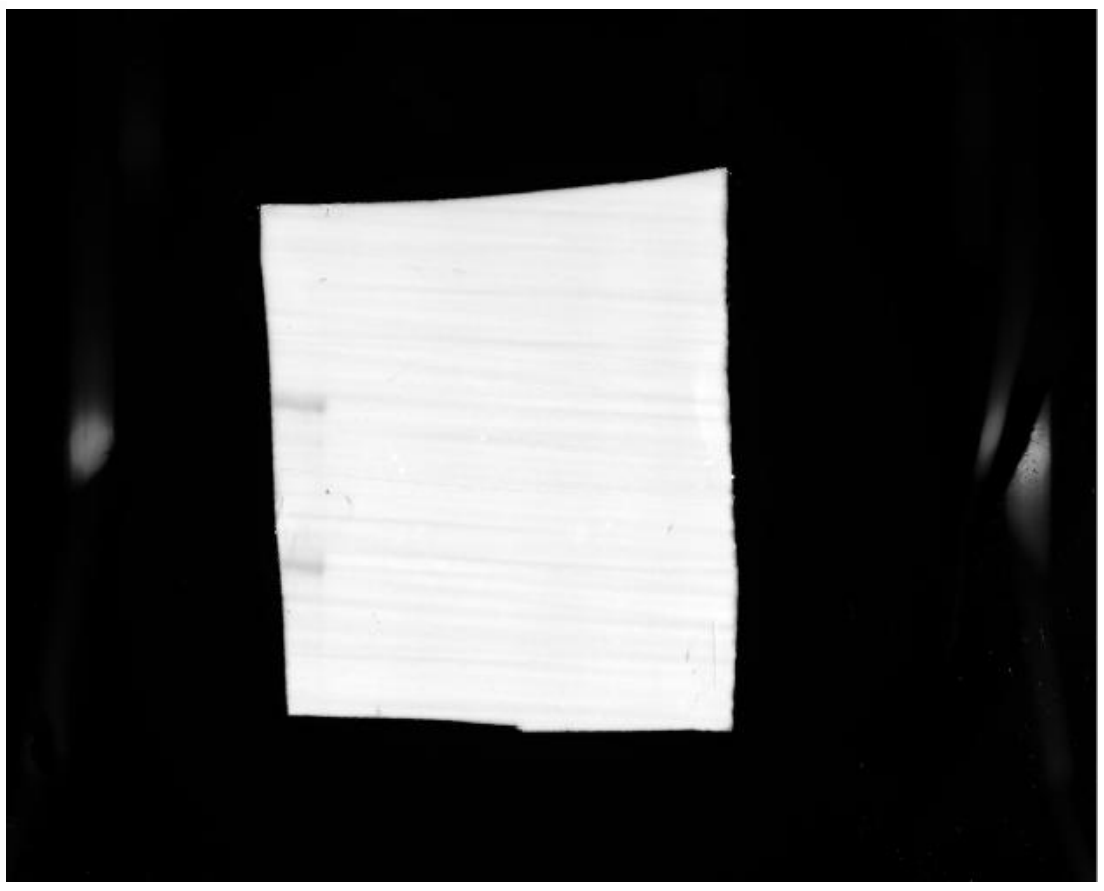

Fig.1i

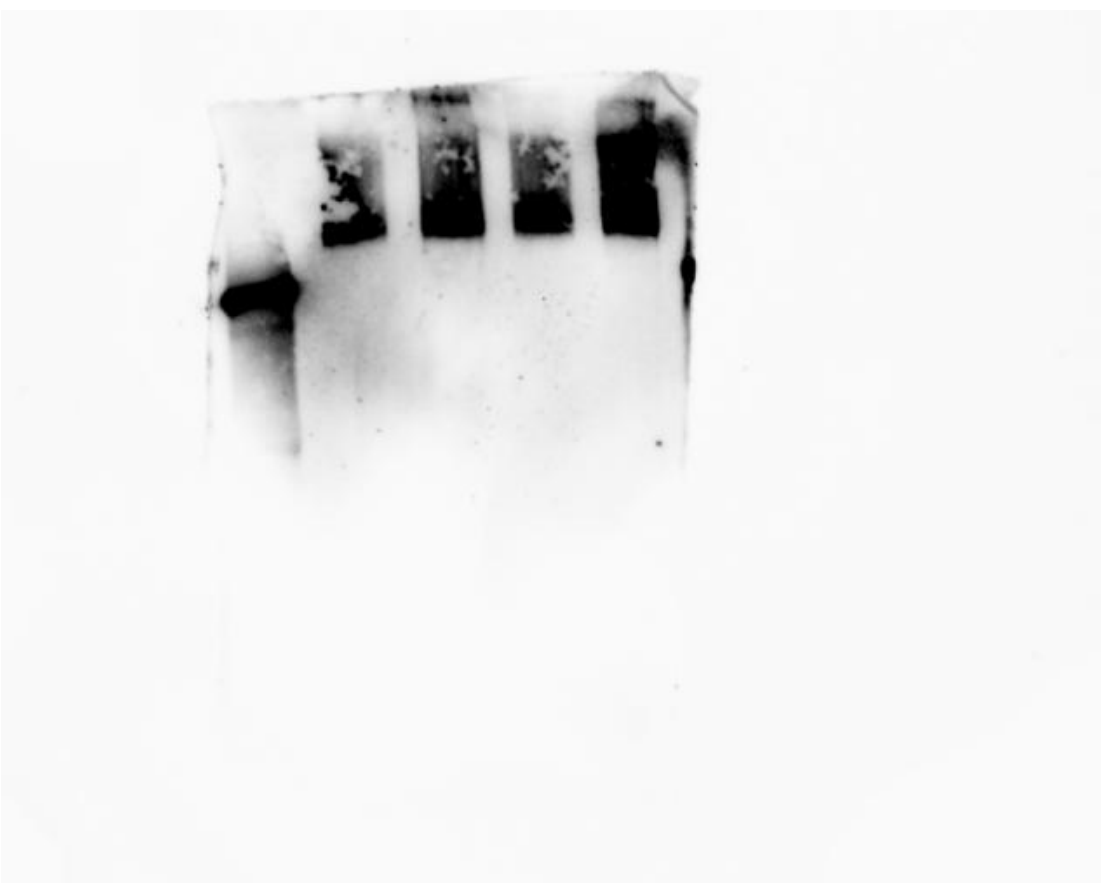

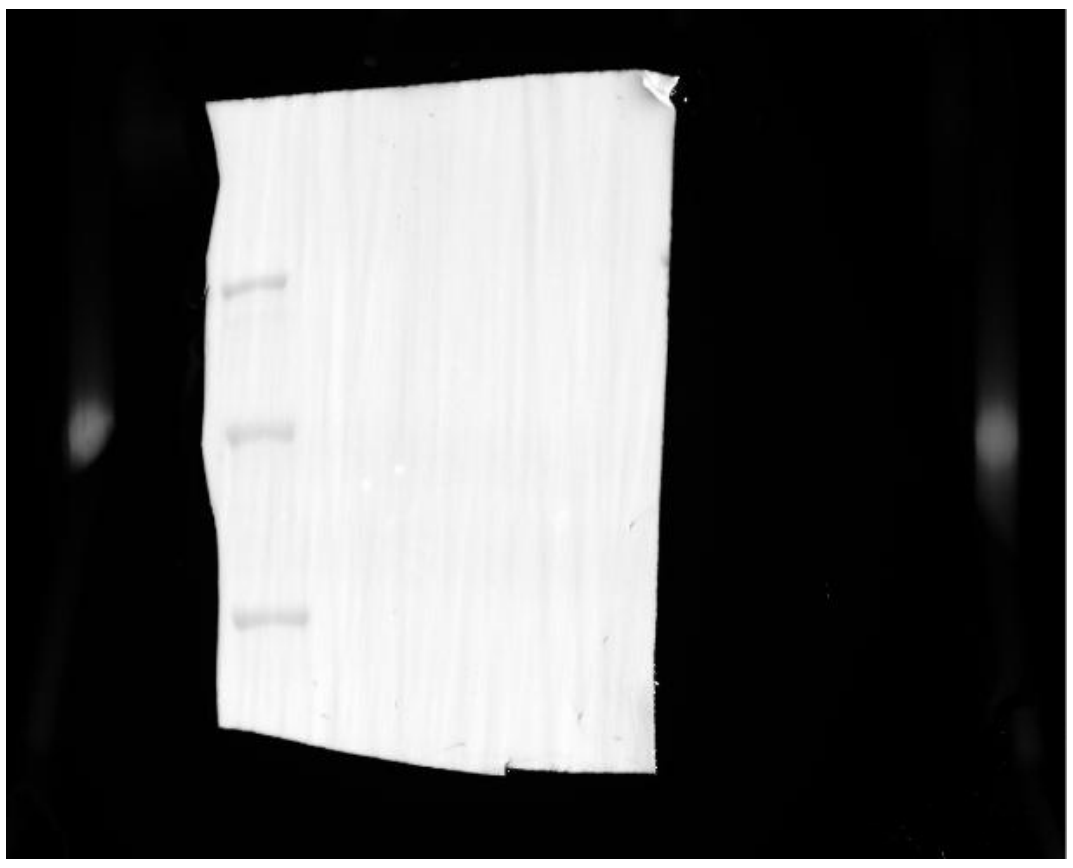
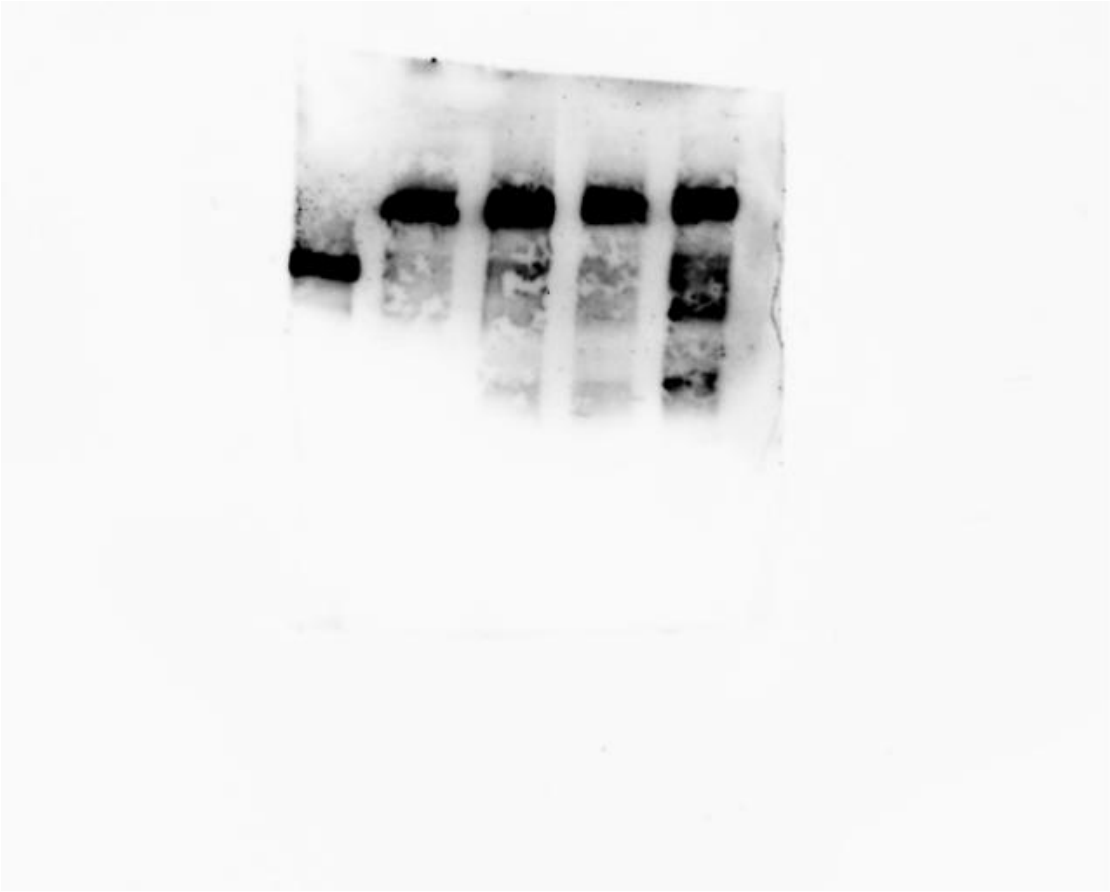
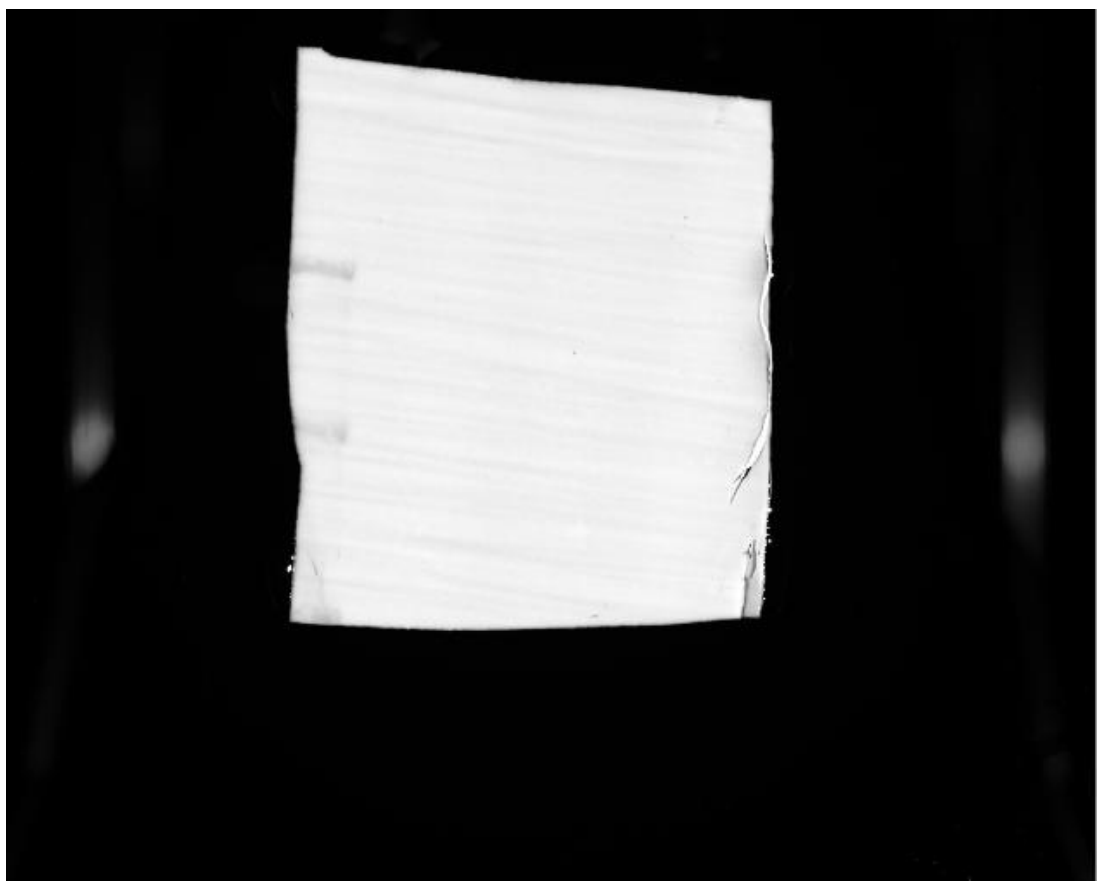

Fig.3d

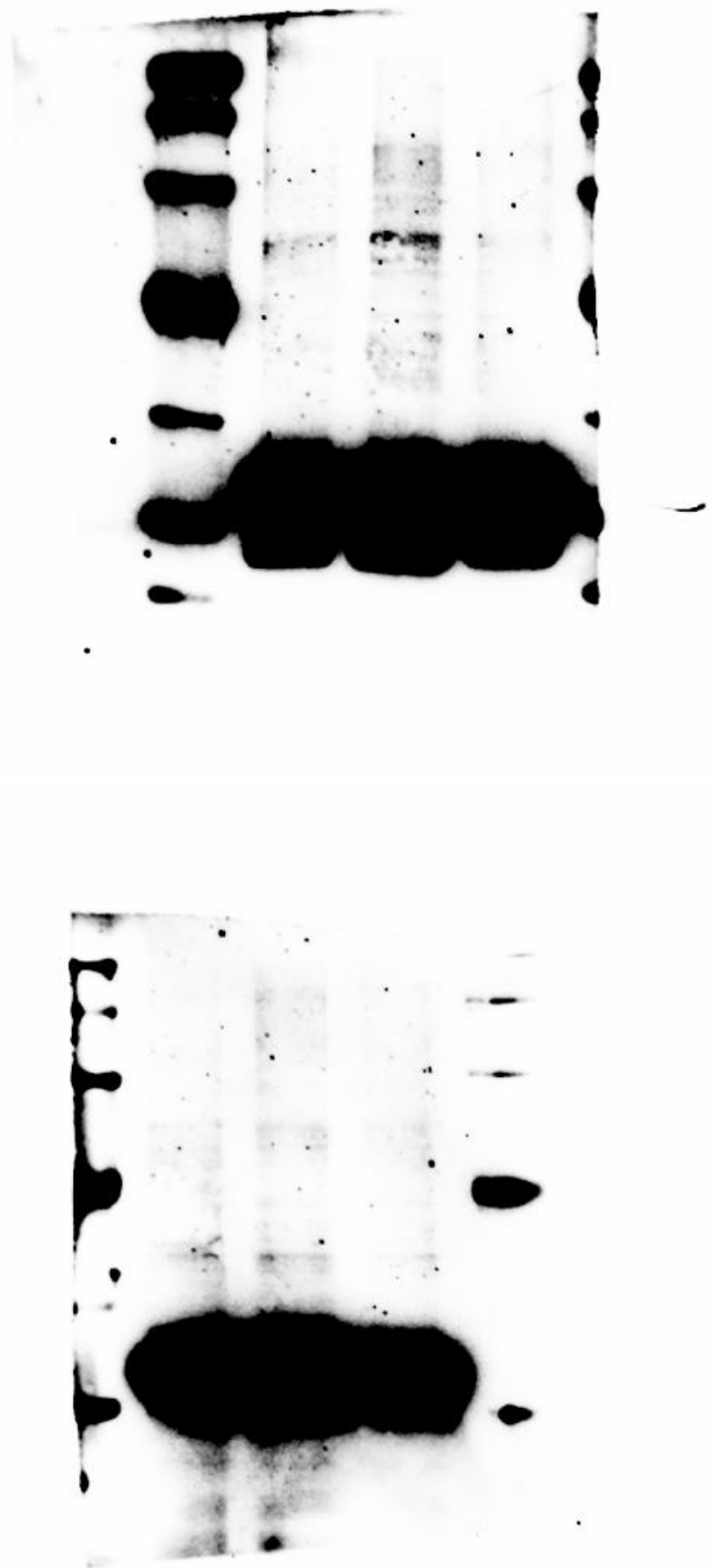

Fig.2e

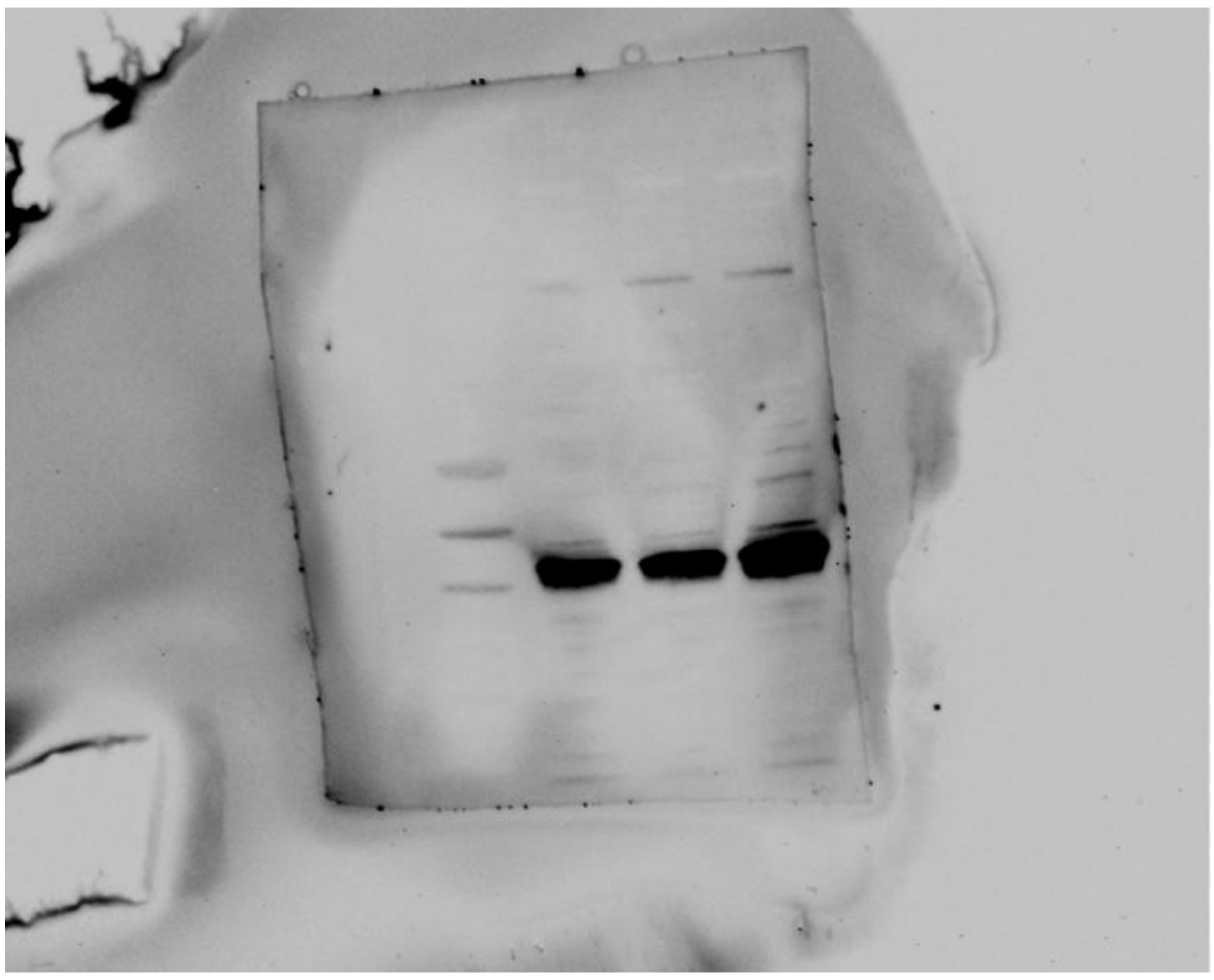

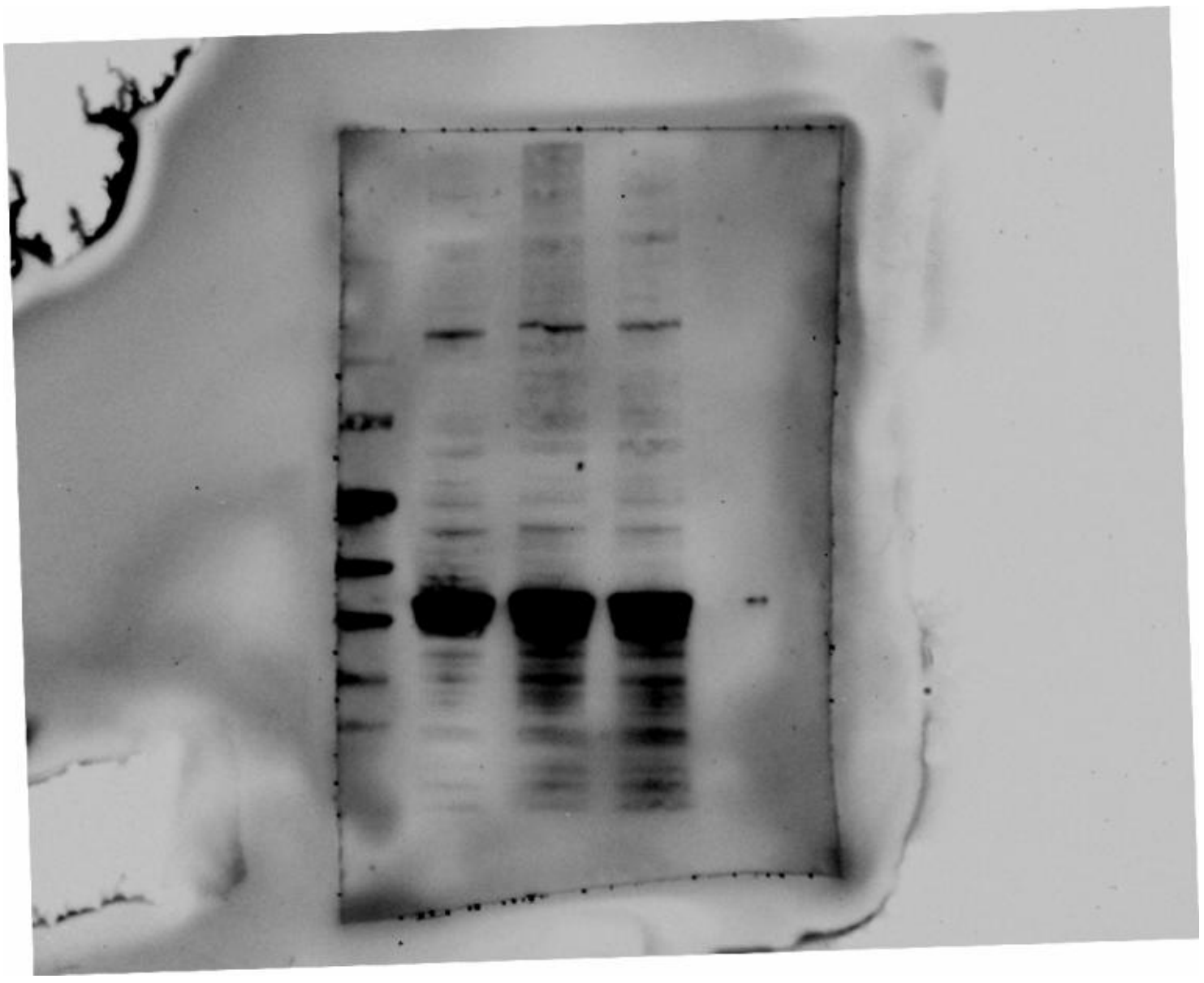

Fig.3g

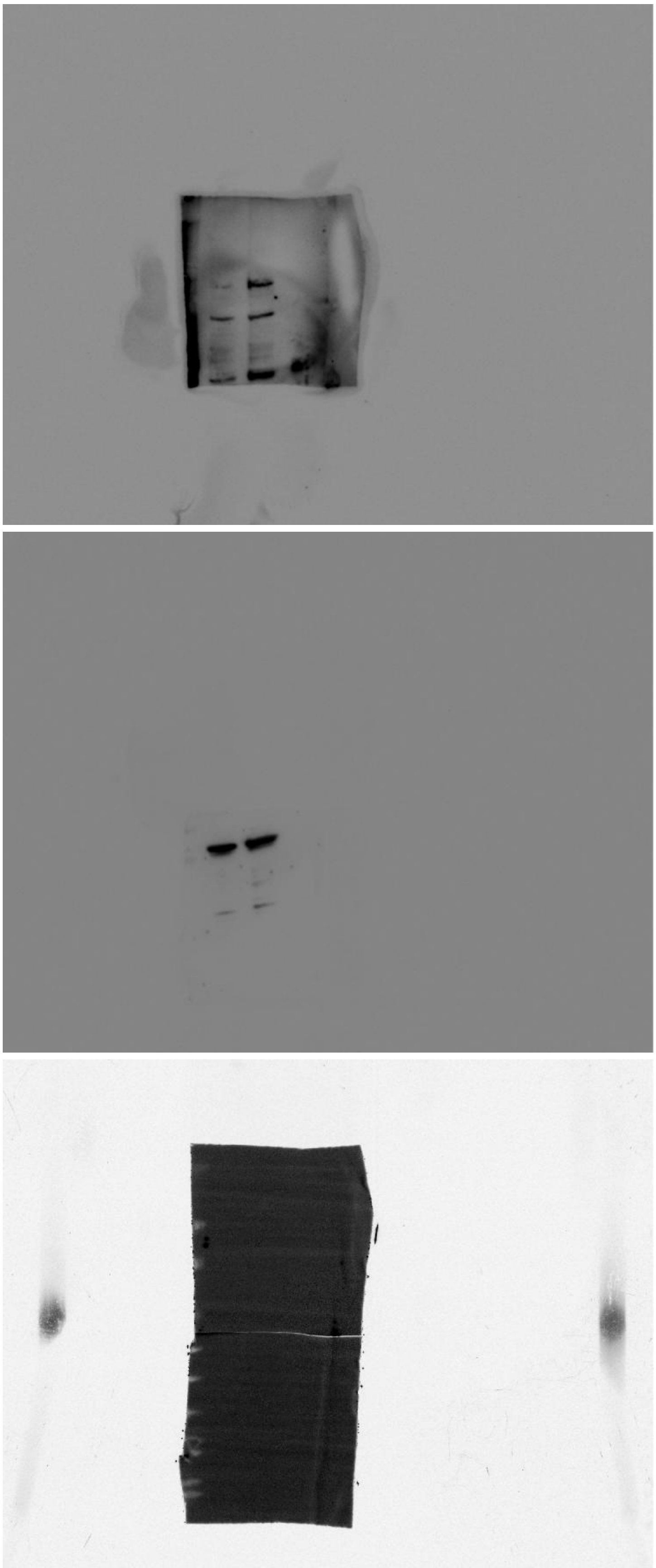

Fig.3k

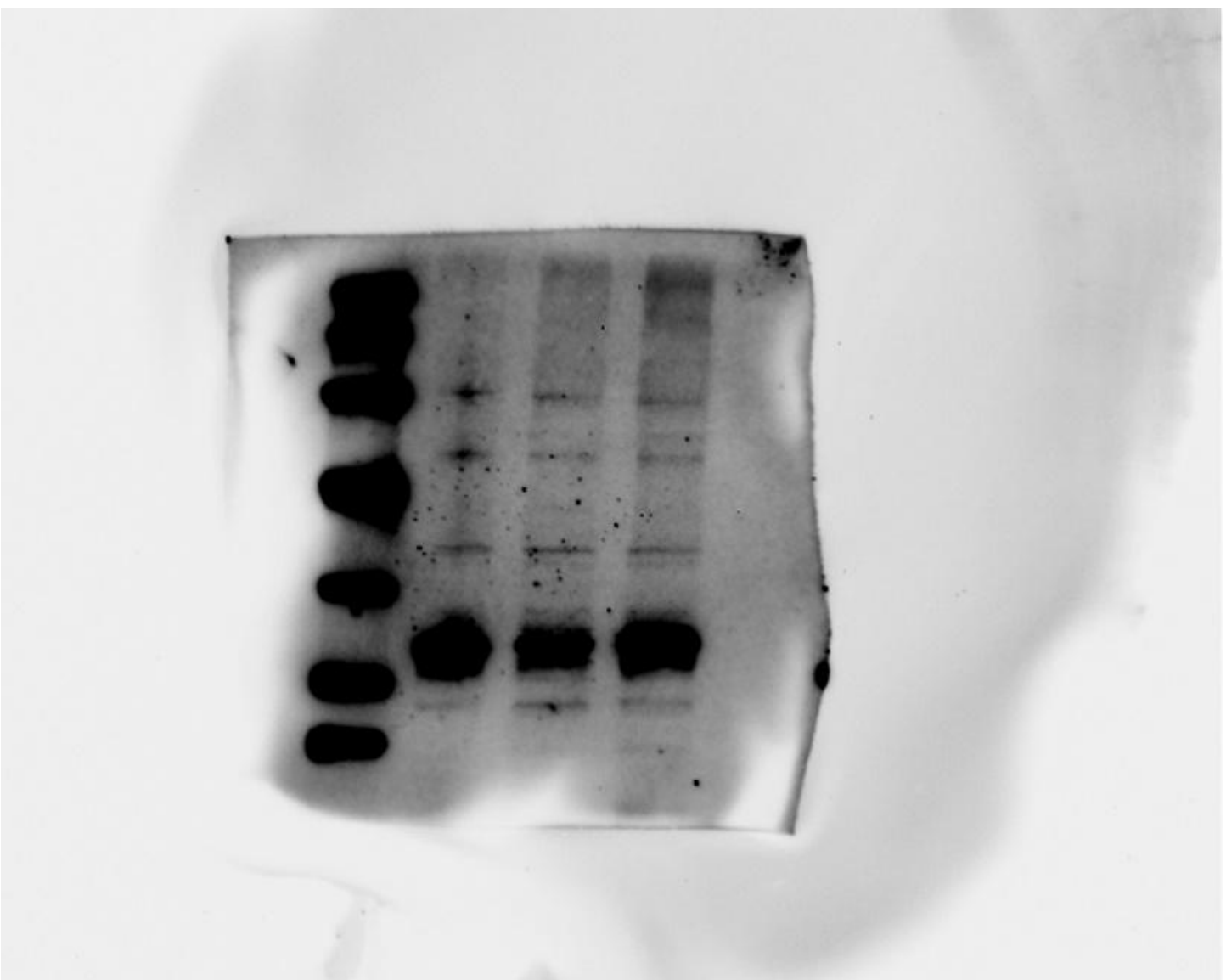

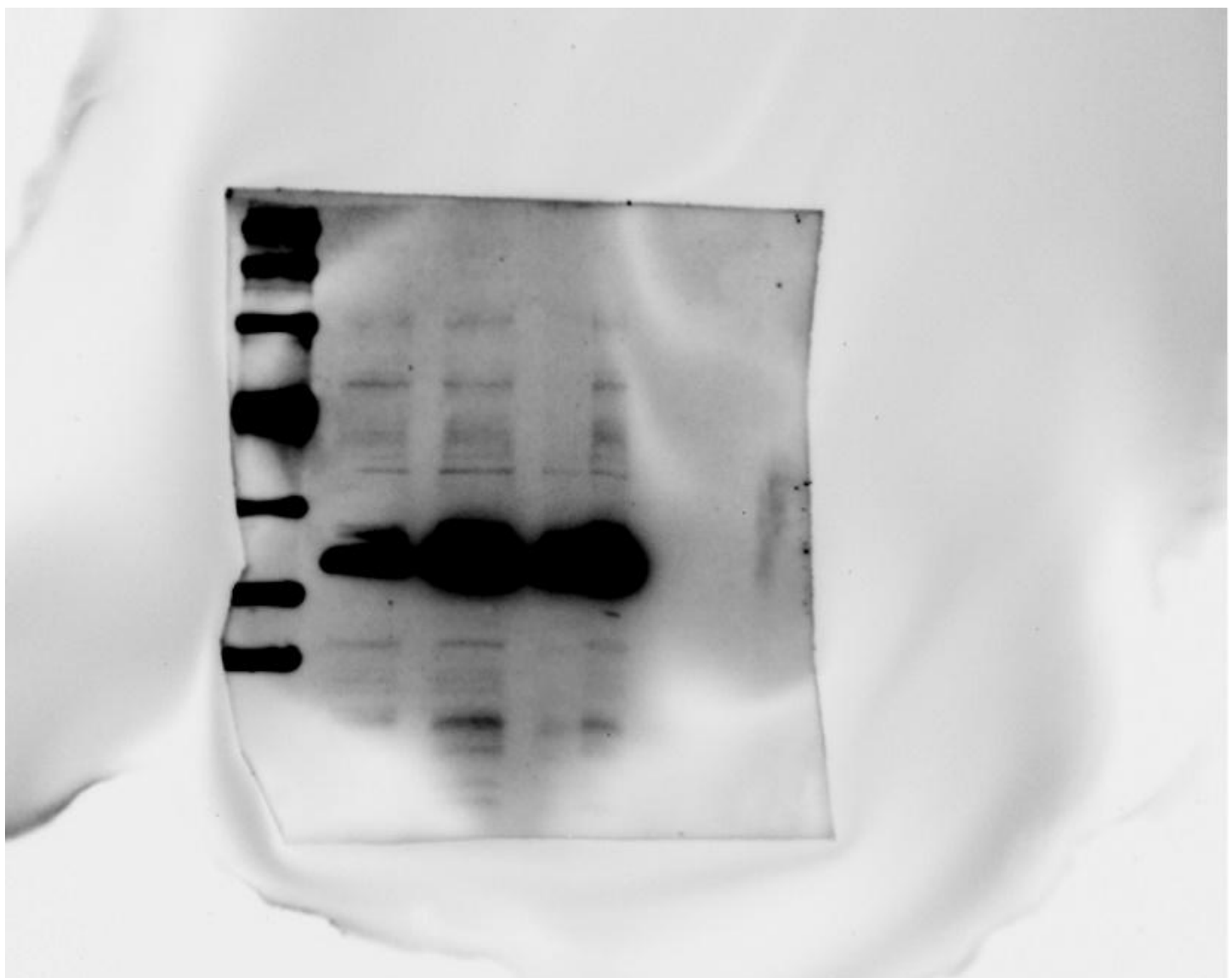

Fig.4a

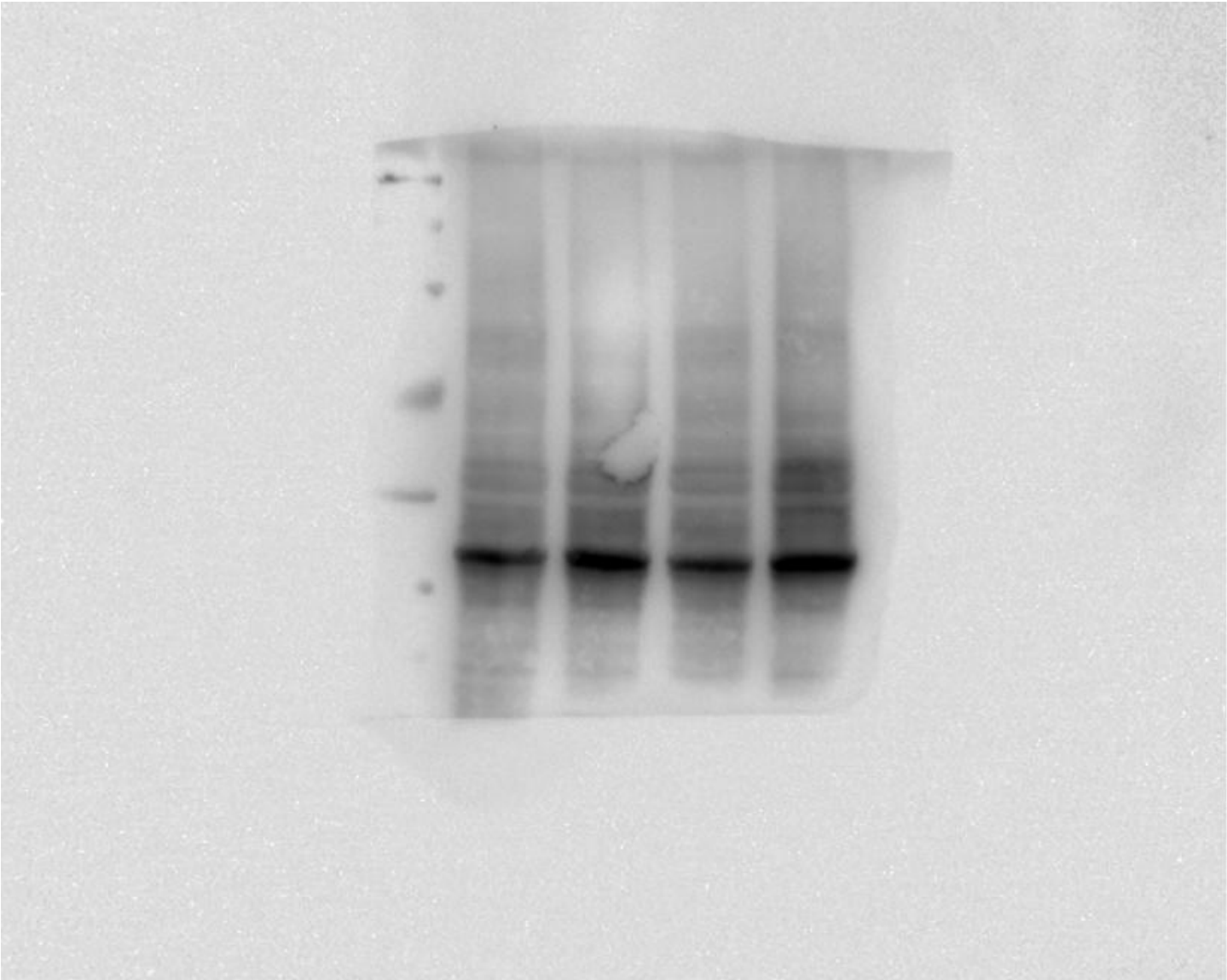

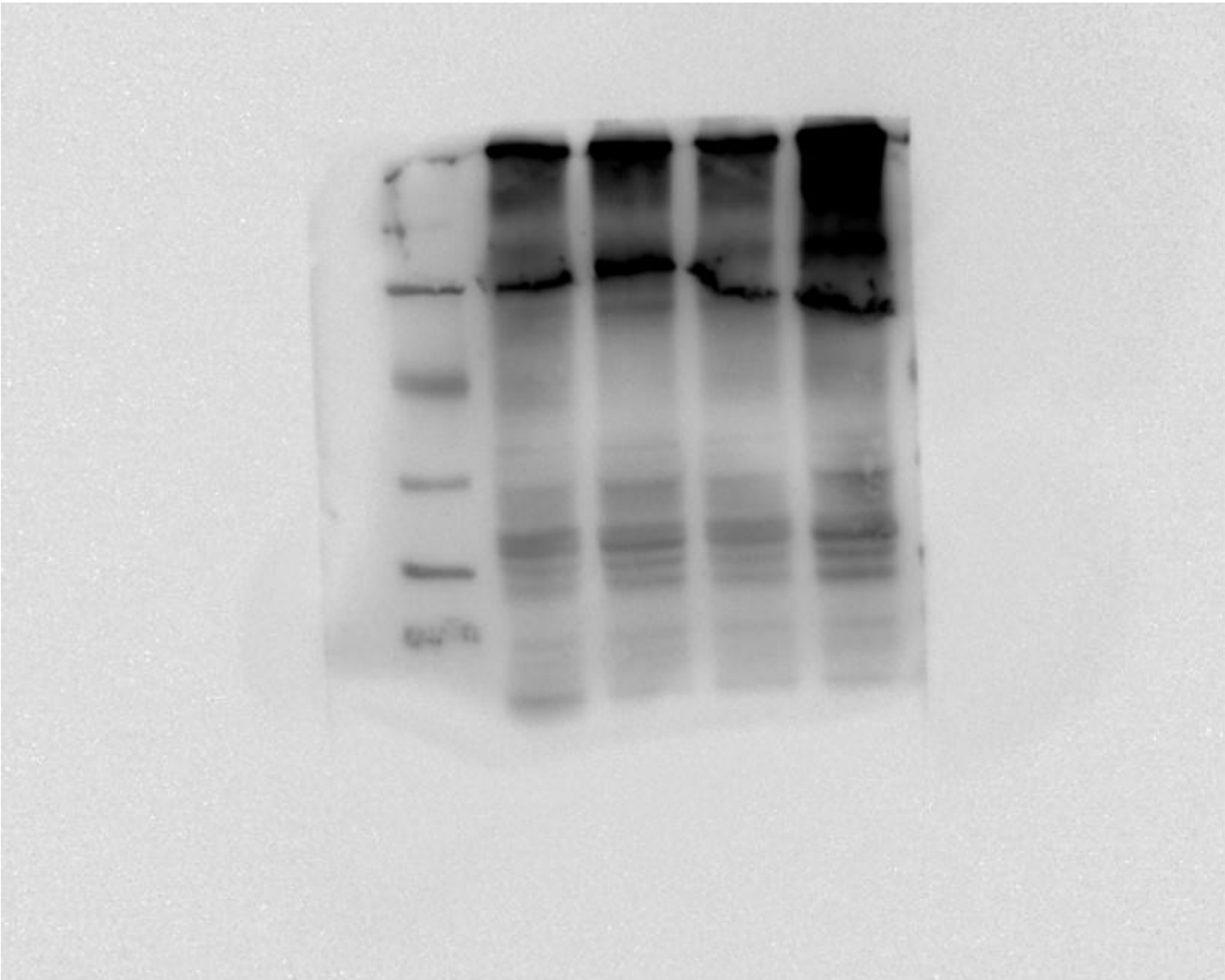

Fig.4c

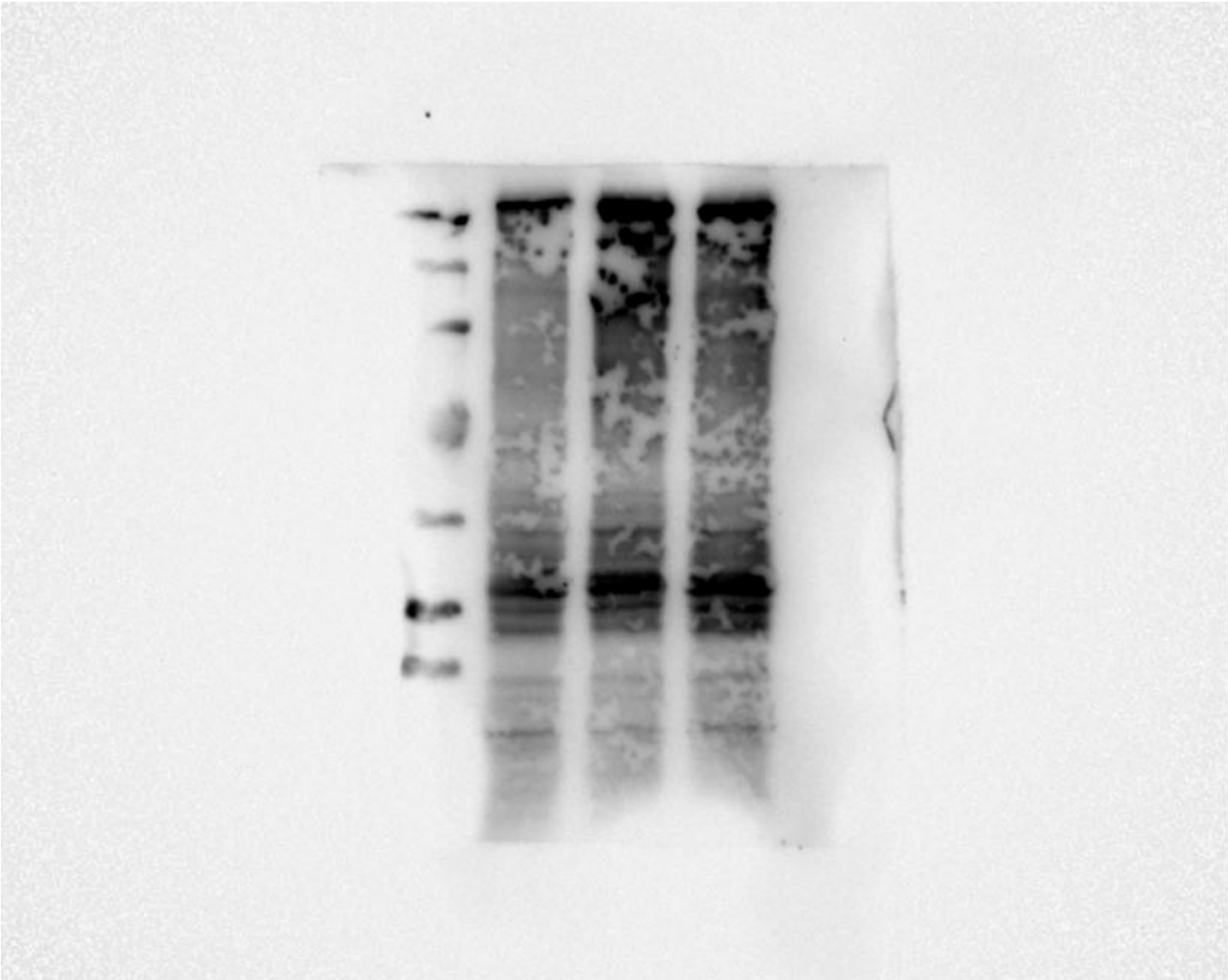

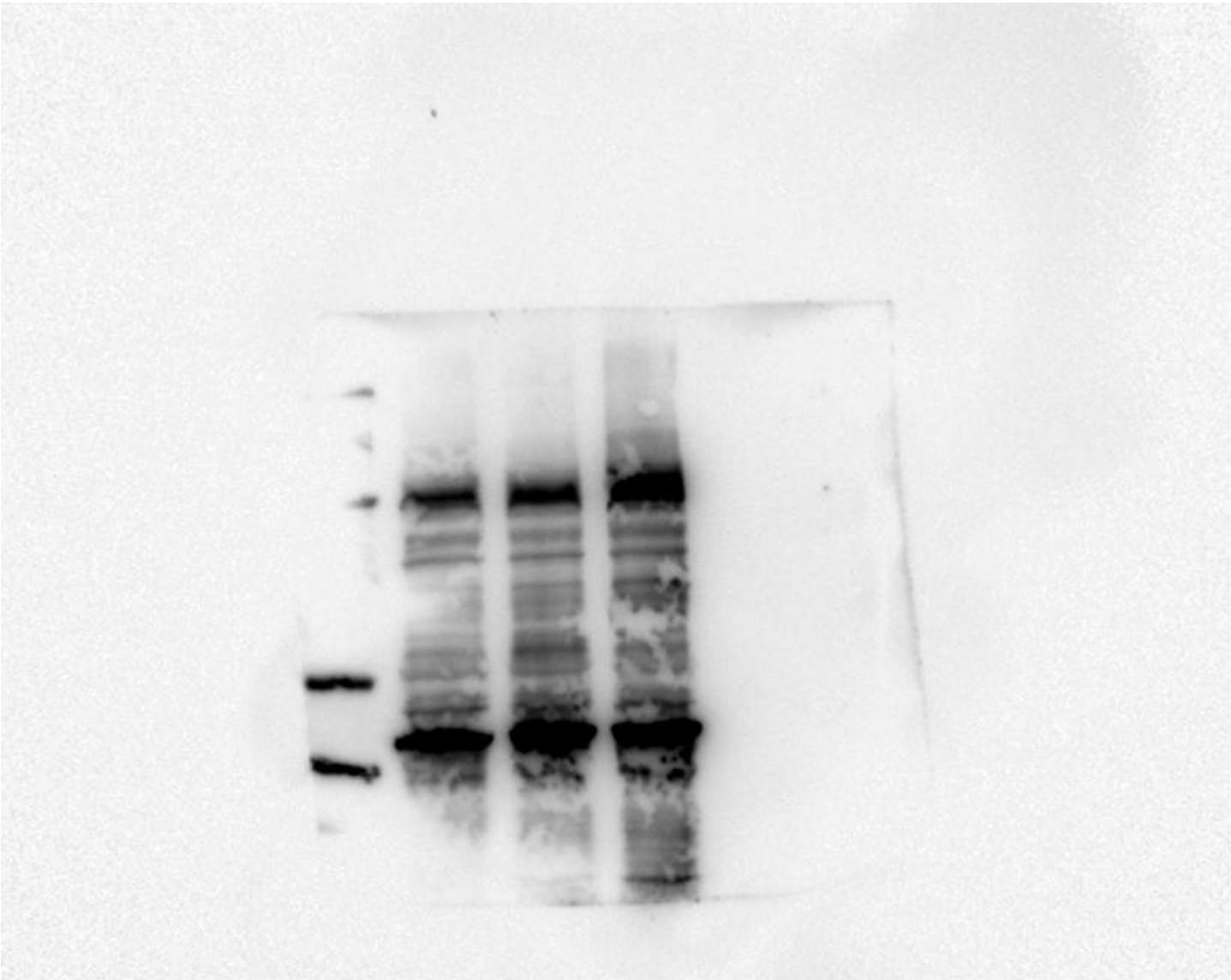

Fig.6g

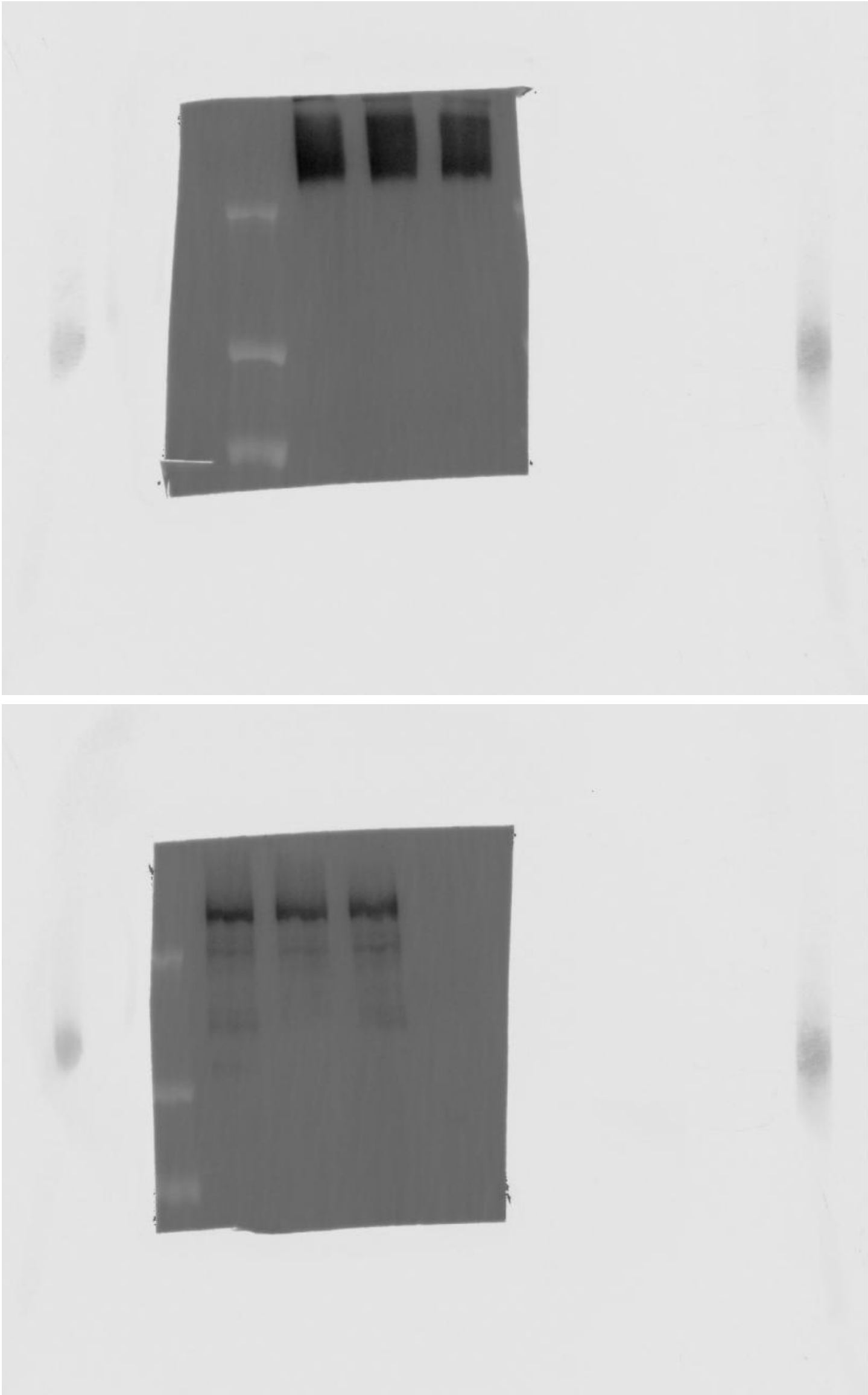

Fig.7h

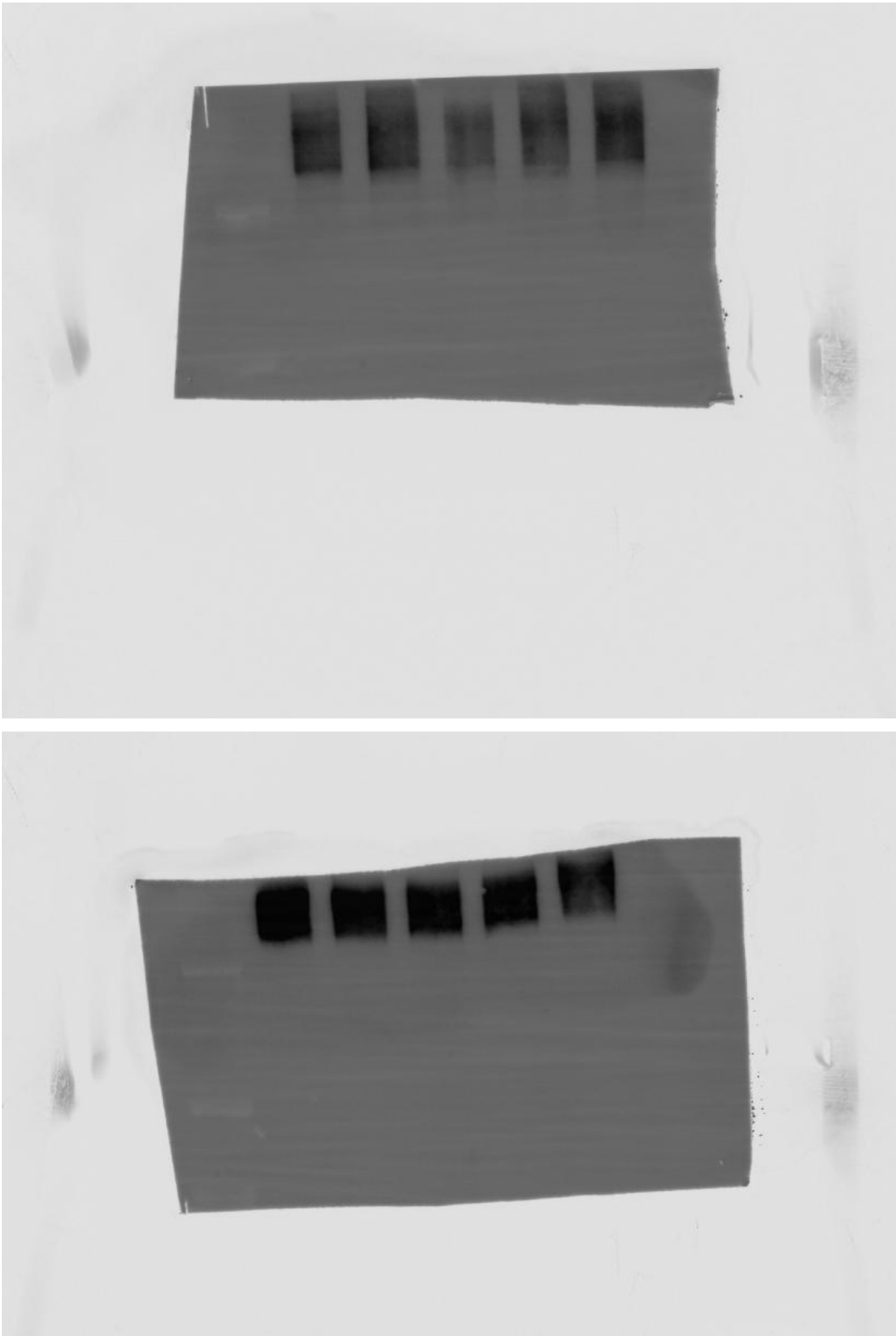

Fig.9b

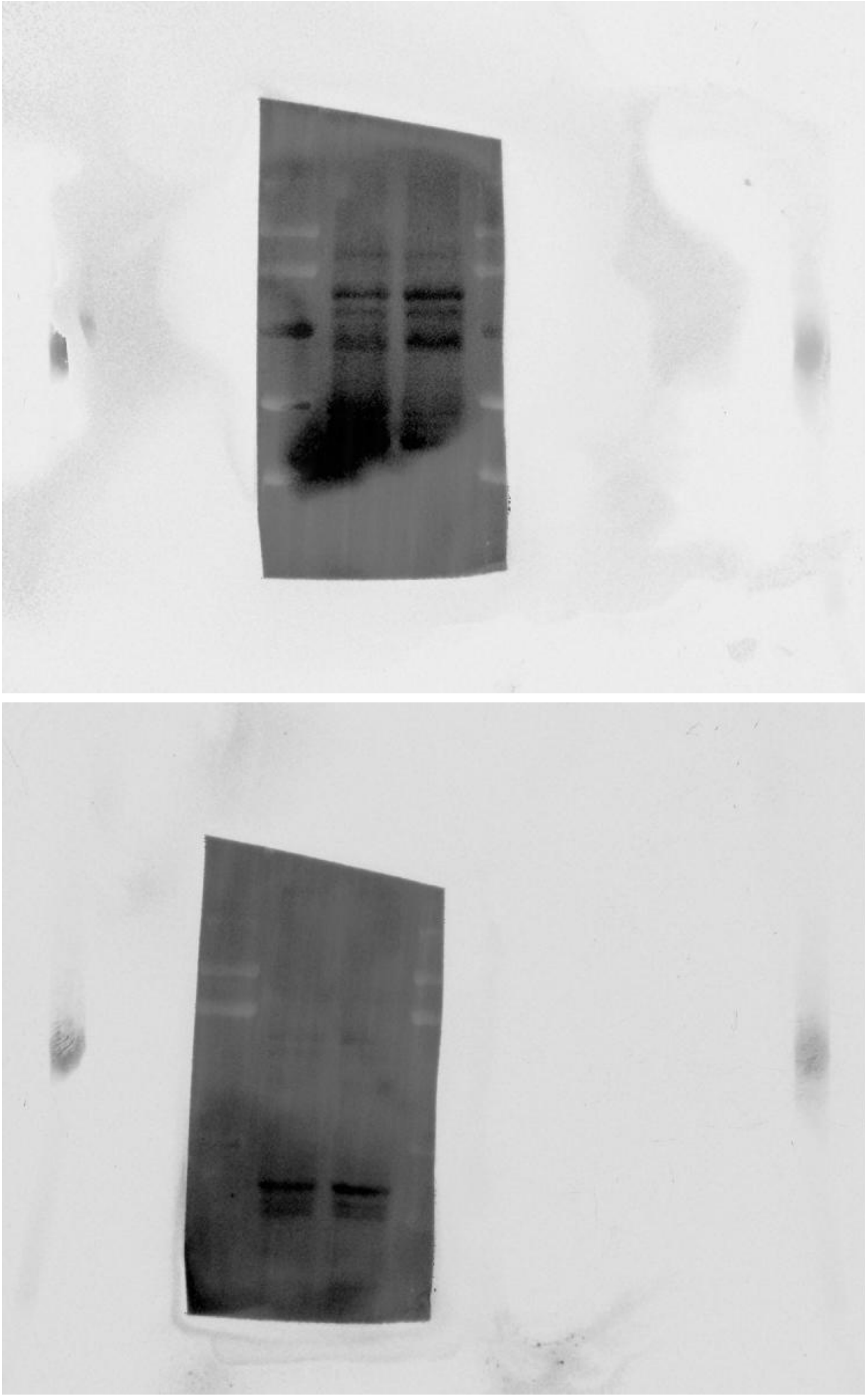

Fig.9l

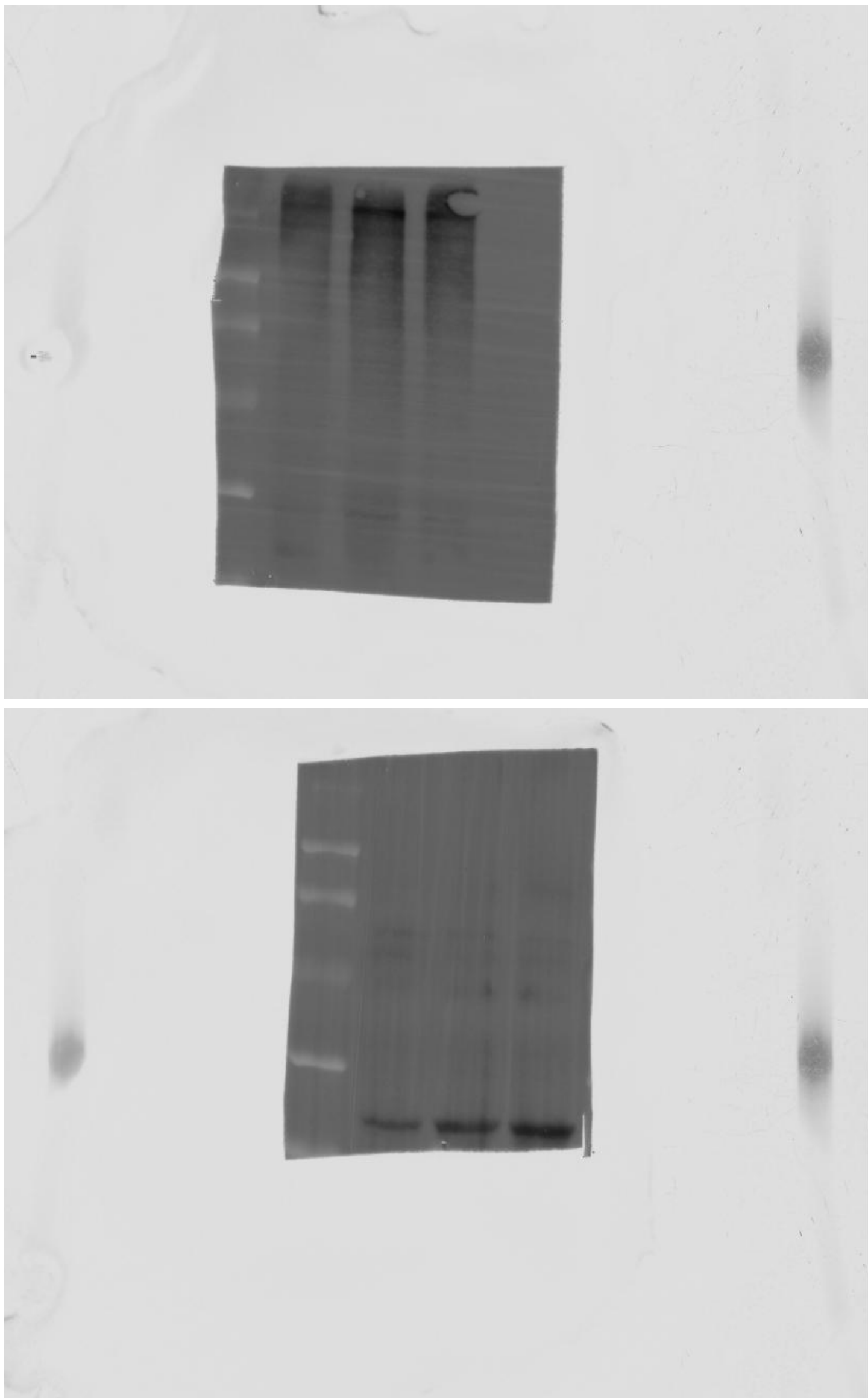

Supplement: Supplementary file 1 [file 41420_2026_3200_MOESM1_ESM.pdf]
