# Supplementary material for: Thioredoxin system dysregulation and calpain activation drive myocardial disulfidptosis via pathological disulfide bonds remodeling
Source: Cell Death Discov. 2026 Jun 19;12:350. doi: 10.1038/s41420-026-03200-0 (PMC13521956; doi:10.1038/s41420-026-03200-0)
Supplement: Supplementary file 2 [file 41420_2026_3200_MOESM2_ESM.docx]

Figure 1 Cysteine residues and cysteine-containing peptides were analyzed by LC-MS/MS in Control, OGD (6 h), and OGD (6 h) + MDL 28170 (6.25 μM) groups, with the figure displaying peptides uniquely detected in the OGD group.

Figure 2 Echocardiographic assessment of cardiac function in Sham, MI, and MI+MDL28170 groups at 6 hours. (6 independent replicates)
